# LIN28B induces a differentiation program through CDX2 in colon cancer

**DOI:** 10.1172/jci.insight.140382

**Published:** 2021-05-10

**Authors:** Kensuke Suzuki, Yasunori Masuike, Rei Mizuno, Uma M. Sachdeva, Priya Chatterji, Sarah F. Andres, Wenping Sun, Andres J. Klein-Szanto, Sepideh Besharati, Helen E. Remotti, Michael P. Verzi, Anil K. Rustgi

**Affiliations:** 1 Herbert Irving Comprehensive Cancer Center, Division of Digestive and Liver Disease, Department of Medicine, Vagelos College of Physicians and Surgeons, Columbia University Irving Medical Center, New York, New York, USA.; 2 Department of Surgery, Graduate School of Medicine, Kyoto University, Kyoto, Japan.; 3 Division of Thoracic Surgery, Massachusetts General Hospital, Boston, Massachusetts, USA.; 4 Broad Institute of MIT and Harvard University, Cambridge, Massachusetts, USA.; 5 Papé Family Pediatric Research Institute, Oregon Health & Science University, Portland, Oregon, USA.; 6 Institute for Biomedical informatics, University of Pennsylvania, Philadelphia, Pennsylvania, USA.; 7 Histopathology Facility and Cancer Biology Program, Fox Chase Cancer Center, Philadelphia, Pennsylvania, USA.; 8 Department of Pathology and Cell Biology, Columbia University Medical Center, New York, New York, USA.; 9 Department of Genetics, Human Genetics Institute of New Jersey, Rutgers University, Piscataway, New Jersey, USA.

**Keywords:** Cell Biology, Gastroenterology, Colorectal cancer, Molecular biology

## Abstract

Most colorectal cancers (CRCs) are moderately differentiated or well differentiated, a status that is preserved even in metastatic tumors. However, the molecular mechanisms underlying CRC differentiation remain to be elucidated. Herein, we unravel a potentially novel posttranscriptional regulatory mechanism via a LIN28B/CDX2 signaling axis that plays a critical role in mediating CRC differentiation. Owing to a large number of mRNA targets, the mRNA-binding protein LIN28B has diverse functions in development, metabolism, tissue regeneration, and tumorigenesis. Our RNA-binding protein IP (RIP) assay revealed that LIN28B directly binds CDX2 mRNA, which is a pivotal homeobox transcription factor in normal intestinal epithelial cell identity and differentiation. Furthermore, LIN28B overexpression resulted in enhanced CDX2 expression to promote differentiation in subcutaneous xenograft tumors generated from CRC cells and metastatic tumor colonization through mesenchymal-epithelial transition in CRC liver metastasis mouse models. A ChIP sequence for CDX2 identified α-methylacyl-CoA racemase (AMACR) as a potentially novel transcriptional target of CDX2 in the context of LIN28B overexpression. We also found that AMACR enhanced intestinal alkaline phosphatase activity, which is known as a key component of intestinal differentiation, through the upregulation of butyric acid. Overall, we demonstrated that LIN28B promotes CRC differentiation through the CDX2/AMACR axis.

## Graphical abstract

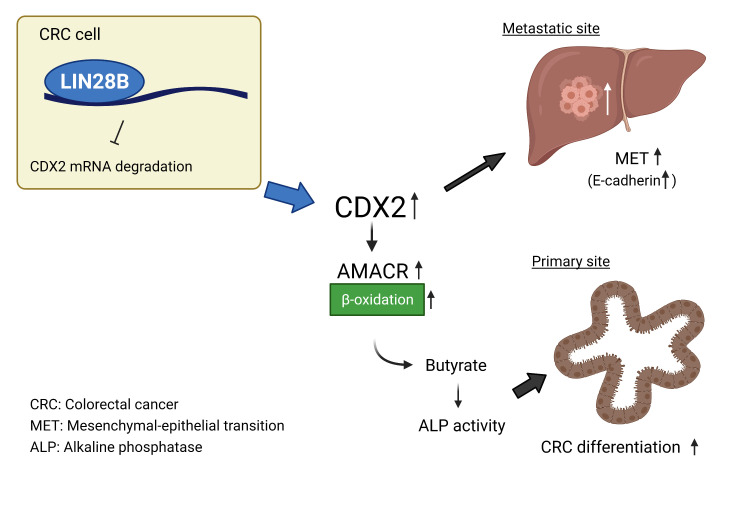

## Introduction

RNA-binding proteins (RBPs), which form mRNA-protein complexes (mRNPs) with their target RNAs, represent key regulators of essential cellular processes (1, 2). Indeed, a number of studies have shown that RBPs, including LIN28B, promote tumorigenesis in diverse cancer types (3–6). We have demonstrated that LIN28B overexpression correlates with poor survival in colon cancers and increased tumor recurrence (7). In addition, we demonstrated previously that LIN28B acts as an oncogene in genetic mouse models of colorectal adenomas and cancers (8, 9), which was corroborated subsequently by others (10). Functionally, as a master regulator of the let-7 family of microRNAs, LIN28B binds pre–/pri–let-7 and blocks their cleavage and maturation, thus upregulating certain oncogenic targets, including c-Myc, HMGA2, and IGF2BP1 (IMP1) (9, 11).

Apart from its role in colorectal cancer (CRC) initiation and progression, there is some evidence that LIN28B might promote CRC differentiation. LIN28B-overexpressing colon cancer cell lines that result in xenograft tumors are more glandular and differentiated compared with xenograft tumors generated from empty vector cell lines (7). Human CRCs with LIN28B overexpression exhibit a differentiated phenotype (11). This also appears consistent with other studies in which LIN28B-overexpressing adenocarcinomas appear more glandular compared with LIN28A-expressing tumors (8, 12–14). However, the mechanism by which LIN28B promotes CRC differentiation remains unclear. Elucidating this axis may not only explain CRC clinical outcomes but also identify potential new therapeutic strategies.

Caudal-related homeobox transcription factor 2 (CDX2) is a critical regulator for intestinal identity and differentiation (15) and its expression is highly specific to the intestinal epithelium (16). Conditional knockout of the murine Cdx2 gene revealed that Cdx2 is essential for the villus morphology and cytodifferentiation of intestinal cells (17, 18). In CRC, histopathological analyses have reported that approximately 90% of CRC tumors express CDX2 (19), and these tumors are associated with well-differentiated or moderately differentiated status (20). Although experimental evidence suggests that CDX2 is considered to be a potential tumor suppressor because it inhibits cell growth, migration, and dissemination of CRC cells (21), CDX2 expression is observed in over 90% of colorectal liver metastases and it is concordant between primary CRC and the corresponding liver metastases (22). These findings imply that differentiated CRC cells with CDX2 expression also have a capacity for metastasis. Yet, the degree of differentiation is not believed to be associated with a proclivity for metastasis (23, 24), and about 96% of colorectal liver metastases exhibit a differentiated morphology (23). Furthermore, differentiation status may not be a prognostic factor in patients with CRC (25). However, the molecular mechanisms underlying CRC differentiation and tumor progression remain elusive.

Previously, we found a substantial number of predicted mRNA binding targets of LIN28B, including CDX2 (8). Therefore, we hypothesized that posttranscriptional regulation of CDX2 by LIN28B may promote CRC differentiation. In the present study, we describe a potentially novel posttranscriptional regulatory pathway mediated via a LIN28B/CDX2 signaling axis as being critical for CRC differentiation. We found that LIN28B bound directly to CDX2 mRNA, induced CDX2 protein expression, and functionally promoted CRC differentiation. Intriguingly, the upregulation of CDX2 by LIN28B inhibited CRC cell invasion in vitro, whereas it promoted metastatic CRC tumor colonization through epithelial-mesenchymal transition (EMT). In addition, we undertook a comprehensive unbiased ChIP-Seq approach and identified α-methylacyl-CoA racemase (AMACR), a mitochondrial and peroxisomal enzyme for beta-oxidation of branched chain fatty acids (26), as a direct downstream target for the LIN28B/CDX2 axis to govern CRC differentiation. Our studies revealed that the upregulated CDX2 promoted CRC differentiation through the induction of AMACR, which mechanistically fostered butyric acid production and intestinal alkaline phosphatase (ALPi) activation. Taken together, these results illustrate the dynamic regulation of CRC differentiation by a LIN28B/CDX2/AMACR axis and help to explain the importance of CRC differentiation.

## Results

### LIN28B upregulates CDX2 expression in colorectal cancer.

**Figure 1:**
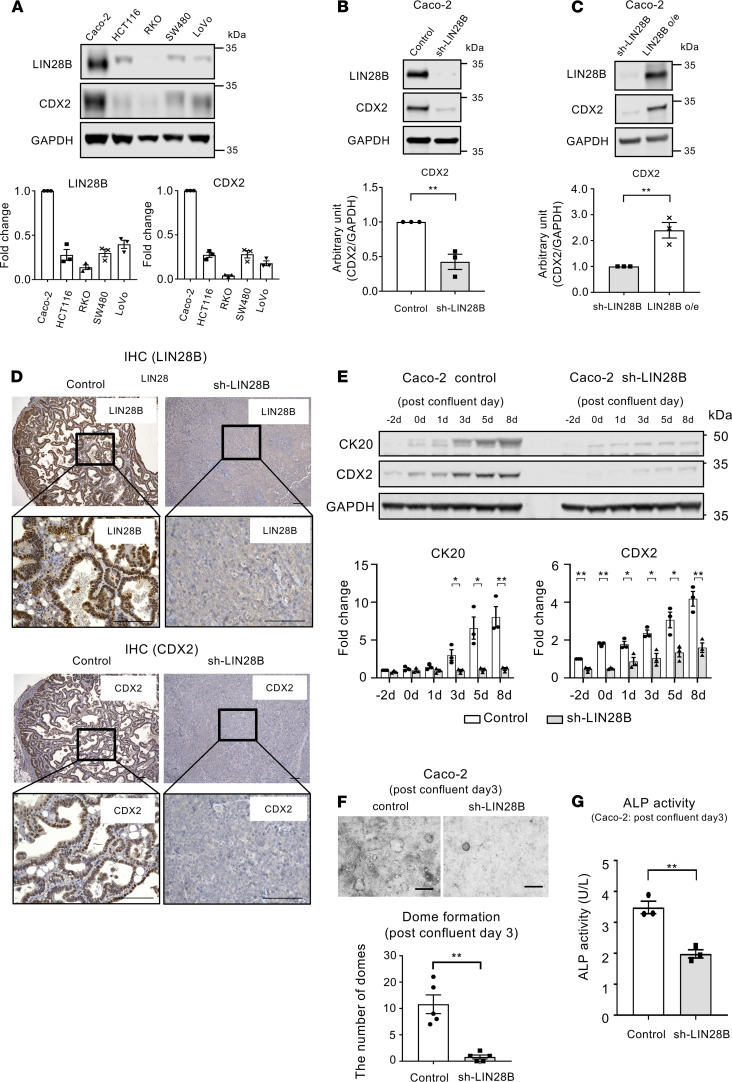
LIN28B upregulates CDX2 expression in colorectal cancer. (**A**) LIN28B and CDX2 expression in human colorectal cancer (CRC) cell lines by Western blotting (WB) analysis. Lower: the fold change of band intensity compared with the expression of target gene in Caco-2 cells (*n* = 3). (**B**) WB analysis of LIN28B and CDX2 in Caco-2 control and LIN28B knockdown (KD) cells. Lower: band intensities were normalized by densitometry to GAPDH (*n* = 3). (**C**) WB analysis of LIN28B and CDX2 in Caco-2 LIN28B KD and LIN28B long isoform expression (LIN28B o/e) cells. Lower: band intensities were normalized by densitometry to GAPDH (*n* = 3). (**D**) Representative IHC staining for LIN28B (left) and CDX2 (right) in the subcutaneous xenograft tumor of Caco-2 cells with control or LIN28B KD. Scale bars: 100 μm. (**E**) Upper: WB analysis of CK20 and CDX2 expression in Caco-2 cells with control or sh-LIN28B at the confluence time point. Lower: densitometry analysis. The value for CK20 or CDX2 at day –2 for the sh-control samples was referred to as 1. (**F**) Upper: representative image for dome formation in Caco-2 cell with control/LIN28B KD at postconfluence day 3. Scale bars: 500 μm. Lower: the graph indicates the number of domes. A dome was defined as greater than 100 μm diameter (*n* = 5). (**G**) ALP activity assay in Caco-2 control and LIN28B KD cells at postconfluence day 3 (*n* = 3). Data are presented as mean ± SEM. Unpaired, 2-tailed Student’s *t* tests were performed. *P < 0.05, **P < 0.01.

CDX2 plays a crucial role in intestinal cell identity and differentiation (15, 17). Given that our published in silico analysis suggested that CDX2 is a predicted mRNA target of LIN28B (8, 11), we hypothesized that posttranscriptional regulation of CDX2 by LIN28B may promote differentiation in LIN28B-overexpressing CRC tumors. To that end, we investigated first LIN28B and CDX2 levels in several human CRC cell lines. As shown in Figure 1A, there was a positive correlation between LIN28B expression and CDX2 expression in these CRC cell lines. Intriguingly, Caco-2 cells, which have a high intrinsic differentiation capacity (27), harbored high endogenous LIN28B and CDX2 expression. Next, we analyzed CDX2 expression in Caco-2 cells with and without shRNA-mediated LIN28B knockdown (28). As shown in Figure 1B, Western blotting revealed that LIN28B knockdown resulted in decreased CDX2 expression. Previously, we generated LIN28B long and short isoform–expressing Caco-2 cells by transfecting these plasmids into Caco-2 LIN28B knockdown cells and showed the LIN28B long isoform plays a dominant role in tumorigenesis (28). Therefore, we evaluated CDX2 expression in LIN28B long isoform–expressing Caco-2 cells and LIN28B knockdown Caco-2 cells. We found that LIN28B long isoform overexpression was accompanied by increased CDX2 protein expression (Figure 1C). Additionally, these results were corroborated in constitutively expressing LIN28B SW480, LoVo cells, and DLD1 cells (Supplemental Figure 1A; supplemental material available online with this article; https://doi.org/10.1172/jci.insight.140382DS1).

We next performed subcutaneous xenograft experiments with CRC cells with and without LIN28B overexpression to evaluate the relationship between LIN28B and CDX2 in vivo. LIN28B knockdown tumors exhibited a less differentiated phenotype compared with controls (Figure 1D and Supplemental Figure 1B). IHC staining revealed that LIN28B-expressing differentiated tumors had higher CDX2 expression than LIN28B knockdown poorly differentiated tumors (Figure 1D and Supplemental Figure 1B).

Caco-2 cells have also served as a useful in vitro model to ascertain the mechanisms contributing to intestinal differentiation. After reaching confluence, these cells cease to proliferate and spontaneously differentiate, as noted by the expression of intestinal differentiation markers and dome formation (29, 30). As shown in Figure 1E, CDX2 expression in Caco-2 control cells was upregulated significantly in comparison to Caco-2 cells with LIN28B knockdown in preconfluent and postconfluent states. Of note, 3 days after confluence, increased CDX2 expression was accompanied by increased cytokeratin 20 (CK20) expression, which is known to be a common CRC differentiation marker (31, 32). We also observed that dome formation in Caco-2 control cells was significantly greater than in LIN28B knockdown Caco-2 cells, consistent with impairment of the differentiation program (Figure 1F). We next measured alkaline phosphatase (ALP) activity in Caco-2 cells with/without LIN28B knockdown because ALP activity has been reported to induce differentiation in the enterocyte lineage toward the colonocyte lineage (33, 34). As shown in Figure 1G, ALP activity in Caco-2 cells with LIN28B knockdown was significantly lower compared with that in Caco-2 control cells at day 3 after confluence, whereas there was no significant difference between the 2 groups in subconfluent Caco-2 cells (Supplemental Figure 1C), suggesting that LIN28B may accelerate CRC tumor differentiation through ALP activation.

### LIN28B stabilizes CDX2 mRNA through direct binding.

**Figure 2:**
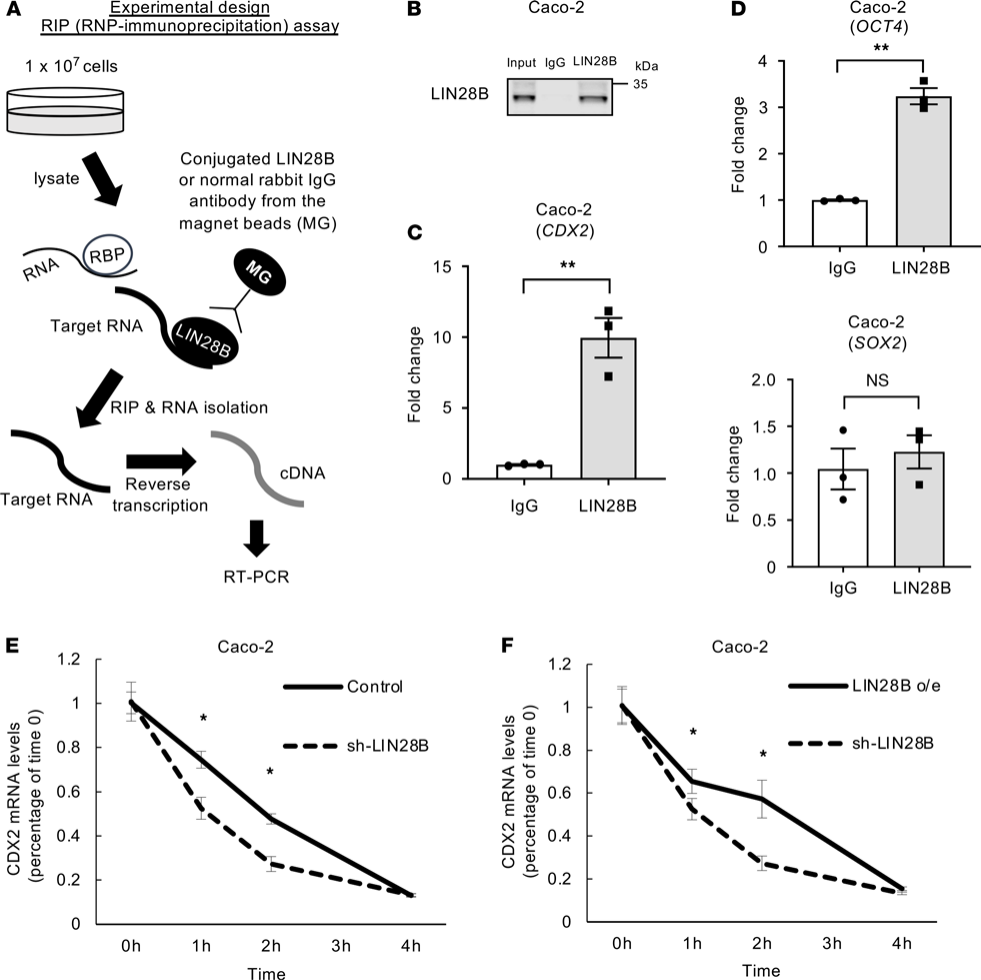
LIN28B stabilized CDX2 mRNA through direct binding. (**A**) Experimental design of RIP assay. (**B**) Quality check of RNA immunoprecipitation (RIP) assay: analysis of LIN28B expression level by WB. The results of RIP assay by qRT-PCR of RIP materials for CDX2 (*n* = 3) (**C**), OCT4 (*n* = 3) (**D**; upper), and SOX2 (*n* = 3) (**D**; lower). mRNA stability assay in Caco-2 control and LIN28B KD cells (**E**) and in Caco-2 LIN28B KD and o/e cells (**F**) (*n* = 3). Data are presented as mean ± SEM. Unpaired, 2-tailed Student’s *t* tests were performed. *P < 0.05, **P < 0.01.

We addressed next how LIN28B upregulates CDX2 in CRC cells. LIN28B is a well-known RBP. RBPs play crucial roles in the regulation of several essential cellular processes, such as RNA splicing, localization, stability, degradation, and translation by binding to the mRNA of target genes (4). LIN28B has also been reported as a regulator of these processes (35). To accomplish this, we first performed an RBP IP (RIP) assay. RBPs stabilize mRNAs, and although this does not typically lead to increased mRNA expression, it does result in increased in protein expression. As shown in Figure 2A (experimental design), we extracted RNA that was bound by LIN28B from Caco-2 cells. We evaluated the quality of these experiments by Western blotting. The target ribonucleoprotein (RNP) complex was successfully concentrated because no LIN28B was detected in the post-IP beads coated with normal rabbit IgG. However, LIN28B was detected in the post-IP beads coated with an anti-LIN28B antibody (Figure 2B). Subsequently, we found that the *CDX2* mRNA obtained from the anti-LIN28B antibody-immunoprecipitants was enriched significantly in Caco-2 cells in comparison with the *CDX2* mRNA isolated from the normal rabbit IgG complex (Figure 2C). A previous study demonstrated that *OCT4* mRNA but not *SOX2* mRNA is a direct target for LIN28 in human embryonic stem cells (36); as positive/negative controls, we verified that LIN28B bound to *OCT4* mRNA but did not bind to *SOX2* mRNA (Figure 2D). Additionally, we corroborated these findings in LoVo cells (Supplemental Figure 2, A–C).

We next measured the stability of *CDX2* mRNA in CRC cells with and without LIN28B expression. We first treated LIN28B knockdown Caco-2 cells and empty vector Caco-2 cells with actinomycin D, an inhibitor of RNA polymerase elongation. After transcription was inhibited, the decay rate of *CDX2* mRNA was measured in the presence or absence of LIN28B. We observed a faster rate of decay in LIN28B knockdown cells than in control empty vector cells (Figure 2E). Furthermore, when LIN28B long isoform–overexpressing Caco-2 cells were treated with actinomycin D, we found that long isoform–overexpressing LIN28B rescued the attenuation of *CDX2* mRNA stability induced by LIN28B knockdown (Figure 2F). We also observed that LIN28B increased *CDX2* mRNA stability in LoVo cells (Supplemental Figure 2D). Taken together, these results suggest that LIN28B can contribute to the stabilization of *CDX2* mRNA through direct binding.

### CDX2 expression is maintained during tumorigenesis in intestine-specific Lin28b-expressing transgenic mice.

To assess the pattern of CDX2 expression during LIN28B-mediated tumorigenesis, we investigated the expression of CDX2 in adenomas in *Vil-Lin28b* mice by IHC staining. Previously, we demonstrated *Vil-Lin28b* mice developed adenomas and adenocarcinomas (8). All samples were scored blindly (scale of 0–3) by a pathologist. CDX2 expression was maintained in adenomas (Supplemental Figure 3A). Well-differentiated tumors also retained CDX2 expression in adenocarcinomas from *Vil-Lin28b* mice (*n* = 5) (Supplemental Figure 3B). Importantly, these findings are not consistent with previous studies, which demonstrated that CDX2 levels are reduced in colonic polyps of *Apc*^+/–^ mutant mice (37, 38) and in adenocarcinomas of mice treated with azoxymethane, which is a chemical carcinogen (39). As shown in Supplemental Figure 3C, CDX2 expression did not change in the normal intestinal epithelium when comparing WT and *Vil-Lin28b* mice. Thus, under normal homeostatic conditions, Lin28b might not affect CDX2 expression but did influence CDX2 expression during tumorigenesis. Taken together, these findings indicate that Lin28b contributes to the maintenance of CDX2 expression during tumorigenesis.

### CDX2 may facilitate CRC cell differentiation in the context of LIN28B expression.

**Figure 3:**
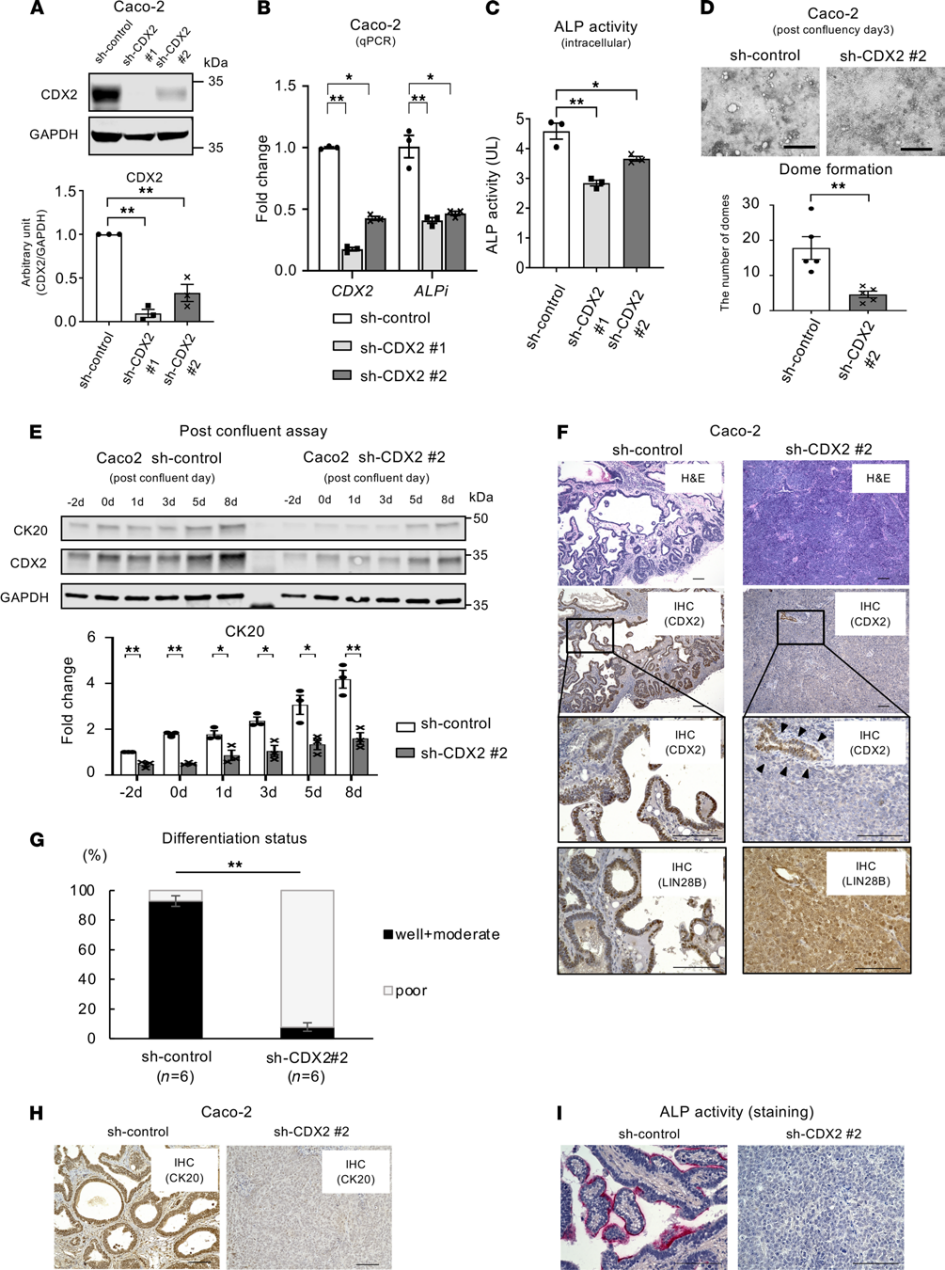
CDX2 regulates CRC tumor differentiation in the context of LIN28B overexpression. (**A**) WB showing CDX2 KD using shCDX2 in Caco2 cells. Lower graph shows the densitometry normalized to GAPDH (*n* = 3). (**B**) *CDX2* and *ALPi* expression (qPCR) in Caco-2 control/CDX2 KD cells (*n* = 3). (**C**) ALP activity assay in Caco-2 control/CDX2 KD cells (*n* = 3). (**D**) Upper: representative image for dome formation in Caco-2 control/CDX2 KD cells at postconfluence day 3. Scale bars: 500 μm. Right: the graph indicates the number of domes. A dome was defined as greater than 100 μm diameter (*n* = 5). (**E**) Upper: WB analysis of CK20 and CDX2 expression in Caco-2 cells with sh-control or sh-CDX2 at the confluence time point. Lower: densitometry. The value for CK20 at day 2 for the sh-control samples was referred to as 1 (*n* = 3). (**F**) Representative IHC in the subcutaneous xenograft tumor of Caco-2 cells with control or CDX2 KD for H&E (first panel), IHC of CDX2 (second and third panels), and IHC of LIN28B (fourth panel). (**G**) Cumulative ratio of differentiation status in subcutaneous xenograft tumors (*n* = 6). (**H**) Representative IHC of CK20 in the subcutaneous xenograft tumor of Caco-2 cells with control or CDX2 KD. (**I**) Representative ALP staining in the subcutaneous xenograft tumor of Caco-2 cells with control or CDX2 KD. Scale bars: 100 μm. Data are presented as mean ± SEM. Unpaired, 2-tailed Student’s *t* test (**A**, **D**, **E**, and **G**) or 1-way ANOVA followed by Dunnett’s multiple-comparison test as post hoc analysis (**B** and **C**) were performed. *P < 0.05, **P < 0.01.

We next evaluated the function of CDX2 in mediating CRC cell differentiation in the context of LIN28B overexpression. We generated CDX2 knockdown in Caco-2 (high endogenous LIN28B expression) and LIN28B-overexpressing LoVo cell lines (Figure 3A and Supplemental Figure 4A). We first performed qPCR analysis to evaluate the expression of ALPi in CDX2 knockdown CRC cells. As shown in Figure 3B and Supplemental Figure 4B, CDX2 knockdown significantly decreased the expression of ALPi. Furthermore, the ALP activity assay revealed that CDX2 knockdown in Caco-2 cells decreased intracellular ALP activity (Figure 3C), suggesting that CDX2 may enhance ALPi activity in the context of LIN28B overexpression. Here, we used only one Caco-2 line, shCDX2 no. 2, as Caco-2 KD cells for the following experiments because we found complete loss of knockdown occurred in Caco-2 shCDX2 no. 1 cells after several passages. It is possible that the near-complete or complete knockdown of CDX2 by shCDX2 no. 1 (Figure 3A) might have led to lethality in Caco-2 cells. Indeed, Cdx2 loss in mice impairs intestinal identity and promotes colonic dysgenesis (15). To further assess the role of CDX2 for CRC differentiation, we performed several in vitro assays with Caco-2 cells. The dome formation analysis revealed that CDX2 knockdown in Caco-2 cells impaired formation of domes, which in turn represent intestinal differentiation (Figure 3D). We also observed that CK20 expression in Caco-2 cells with CDX2 knockdown was downregulated significantly compared with Caco-2 control cells in postconfluent (differentiated) states (Figure 3E). These results strongly suggest LIN28B may promote CRC differentiation through the direct upregulation of CDX2. To verify this premise in an in vivo setting, we next established xenograft tumors by s.c. injecting these cell lines into the rear flanks of athymic nude mice. Of note, we used subconfluent Caco-2-CDX2 shRNA cells because the confluent status may affect tumorigenicity (39). We reported previously that s.c.-injected tumors with LIN28B overexpression cells were more differentiated in comparison to tumors with low LIN28B expression (7). Intriguingly, histopathological examination revealed that CDX2 knockdown in the context of LIN28B overexpression dramatically converted the tumor’s differentiation status from well or moderately differentiated, which have gland-like structures, to poorly differentiated (Figure 3F and Supplemental Figure 4C). Although CDX2 knockdown was effective, it was not complete. As a result, CDX2 expression was maintained in areas of tumor differentiation (Figure 3E, arrowheads). This indicates that CDX2 plays a pivotal role for CRC tumor differentiation. Furthermore, viable tumor areas were scored as being poorly, moderately, and/or well differentiated in a blinded fashion by a pathologist, revealing statistically significant differences between control and CDX2 knockdown xenograft tumors (Figure 3G and Supplemental Figure 4D). Notably, as shown in Supplemental Figure 4, A and C, the functional consequence of CDX2 knockdown was evident in poorly differentiated LoVo xenograft tumors. We found CK20 expression was downregulated in CDX2 knockdown xenograft tumors (Figure 3H and Supplemental Figure 4E). ALP activity was decreased with CDX2 knockdown in Caco-2 cells (Figure 3I). These findings suggest that LIN28B-induced CDX2 upregulation promotes CRC differentiation through the activation of ALPi.

### CDX2 promotes metastatic CRC tumor colonization through mesenchymal-epithelial transition.

**Figure 4:**
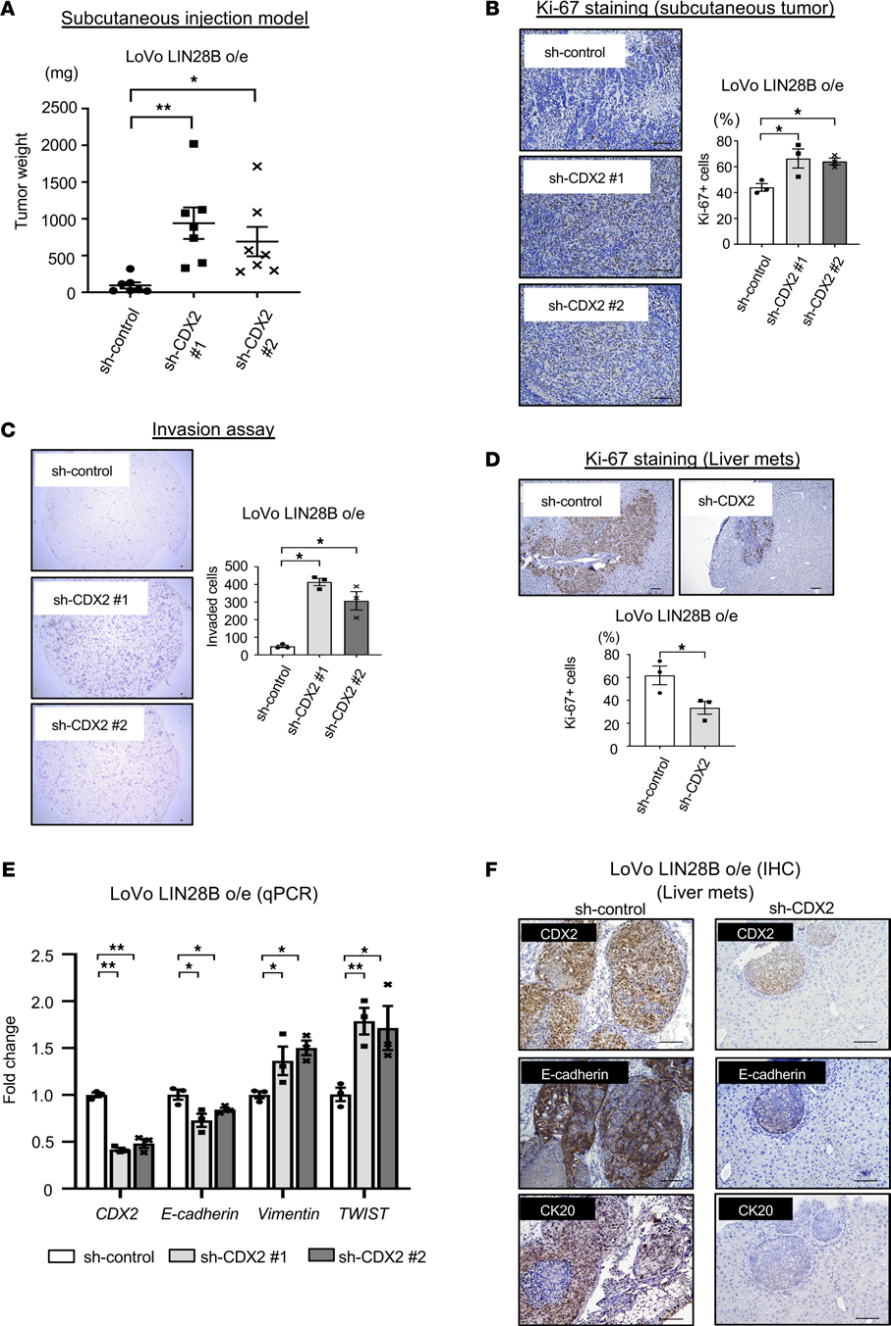
CDX2 promotes metastatic CRC tumor colonization through mesenchymal-epithelial transition. (**A**) Left: subcutaneous xenograft experiments with LoVo cells (*n* = 6 per cell type) showed a significant increase in tumor weight with CDX2 KD with sh-CDX2 no. 1 (472.38 ± 126.23 mg at euthanization) or sh-CDX2 no. 2 (353.38 ± 112.90 mg at euthanization) as compared with control cells (123.88 ± 48.86 mg at euthanization). Right: the images of tumors. Scale bars: 10 mm. (**B**) Ki-67 staining in the subcutaneous xenograft tumor of LoVo cells with control or CDX2 KD, representative images (upper) and the quantification (lower). Scale bars: 100 μm. *n* = 3. (**C**) Transwell chamber invasion assay of LoVo cells with control or CDX2 KD, representative images (upper) and the quantification (lower). Scale bars: 100 μm. *n* = 3. (**D**) Ki-67 staining in the liver metastatic tumor of LoVo cells with control or CDX2 KD, representative images (upper) and the quantification (lower). Scale bars: 100 μm. *n* = 3. (**E**) Epithelial-mesenchymal transition (EMT) marker expression (qPCR) in LoVo control/CDX2 KD cells (*n* = 3). (**F**) Representative IHC of CDX2, E-cadherin, and CK20 in the liver metastatic tumor of LoVo cells with control or CDX2 KD. Scale bars: 100 μm. Data are presented as mean ± SEM. Unpaired, 2-tailed Student’s *t* test (**D**) and 1-way ANOVA followed by Dunnett’s multiple-comparison test as post hoc analysis (**A**, **B**, **C**, and **E**) were performed. *P < 0.05, **P < 0.01.

Given that the majority of primary CRC and corresponding liver metastases retain CDX2 expression (22), primary CRC may progress to metastasis while maintaining a well- to moderately differentiated status. Indeed, we demonstrated previously that differentiated subcutaneous tumors with LIN28B overexpression readily metastasize to the liver compared with less-differentiated tumors without LIN28B expression (7). Therefore, we next focused on how CDX2 can modulate the CRC tumor progression in the context of LIN28B overexpression. We observed that the weights of combined LIN28B overexpression and CDX2 knockdown subcutaneous tumors were increased compared with control tumors (Figure 4A and Supplemental Figure 5A). Additionally, Ki-67 staining revealed that CDX2 knockdown increased cell proliferation in LIN28B-overexpressing CRC cells in subcutaneous tumors (Figure 4B and Supplemental Figure 5B). These findings imply that the balance between proliferation and differentiation may shift toward proliferation with CDX2 knockdown in the context of LIN28B overexpression, and CDX2 might be a critical regulator for the transition from proliferation to differentiation in the subset of CRC cells with LIN28B overexpression.

We next evaluated the metastatic potential of CDX2 in LIN28B-overexpressing CRC cells. Previous studies have reported Caco-2 cells are not optimal for a metastasis assay (40, 41). Therefore, we utilized LIN28B-overexpressing LoVo and DLD1 cells for these analyses. As shown in Figure 4C and Supplemental Figure 5C, the Transwell 2D invasion assays revealed CDX2 knockdown enhanced tumor cell invasion in the context of LIN28B overexpression. To assess metastatic potential in vivo, we used an in vivo liver metastasis assay in which CRC cells are injected directly into the portal vein of mice. Surprisingly, CDX2 knockdown inhibited the growth of metastatic tumors in the liver (Table 1 and Supplemental Figure 5D). Indeed, Ki-67 staining revealed metastatic tumors with CDX2 expression were more proliferative than metastatic tumors with CDX2 knockdown (Figure 4D and Supplemental Figure 5E). To elucidate how CDX2 may promote tumor growth at the metastatic site, we evaluated certain EMT markers in CDX2 knockdown CRC cells because some evidence suggests mesenchymal-epithelial transition (MET) is essential for metastatic tumor colonization in the liver (42, 43). Intriguingly, CDX2 knockdown suppressed the expression of E-cadherin, which is a crucial MET marker, whereas it enhanced the expression of some EMT markers, such as Vimentin and TWIST (Figure 4E and Supplemental Figure 5F). Additionally, IHC staining revealed that metastatic tumors with high CDX2 expression had higher E-cadherin expression in comparison to metastatic tumors with low CDX2 expression (Figure 4F and Supplemental Figure 5G). This is consistent with other studies that have shown CDX2 upregulates E-cadherin expression (44, 45). Moreover, metastatic tumors with CDX2 expression had higher CK20 expression in contrast to metastatic tumors with CDX2 knockdown, which suggests they were more differentiated (Table 2, Figure 4F, and Supplemental Figure 5H). In summary, these findings support the notion that CRC may progress to metastasis while maintaining a differentiated status through MET mediated by CDX2 expression in the background of LIN28B overexpression.

### CDX2 regulates AMACR gene expression in the context of LIN28B high expression.

**Figure 5:**
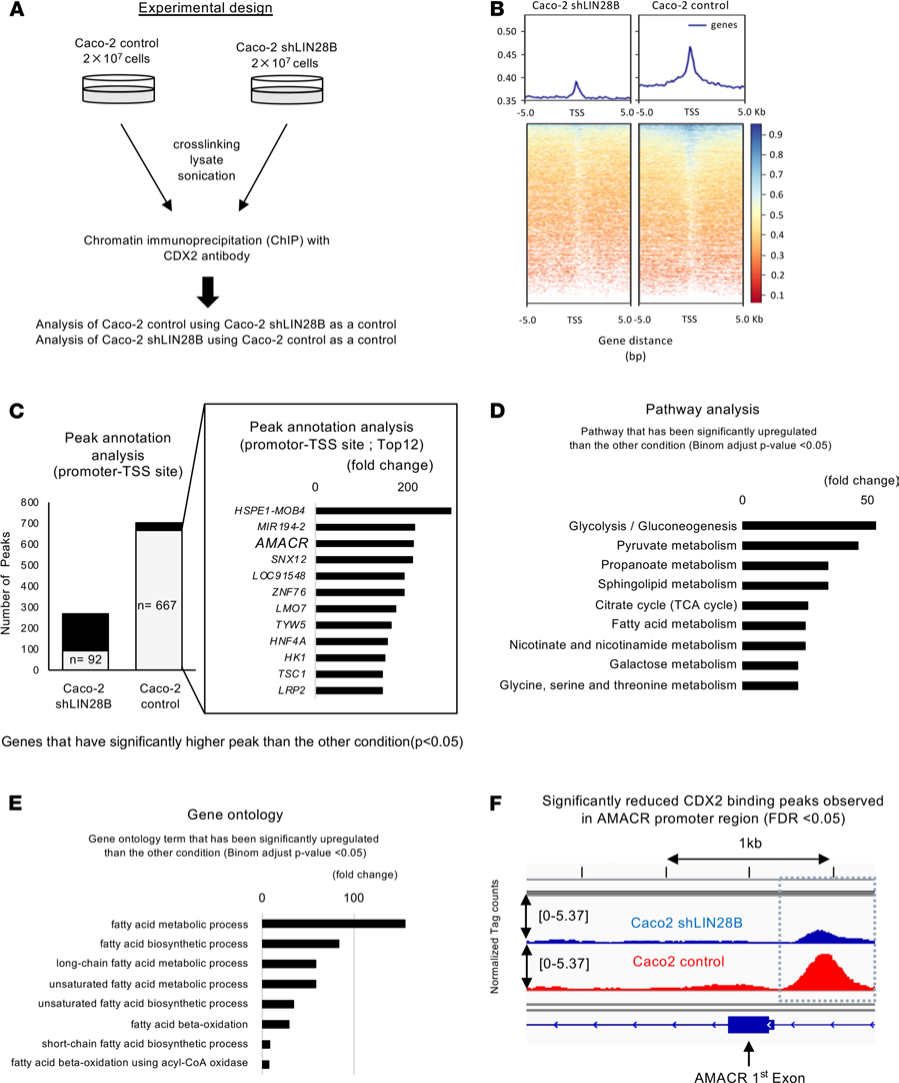
CDX2 ChIP-Seq identifies AMACR as a novel target for the LIN28B/CDX2 axis. (**A**) Experimental design of ChIP-Seq. (**B**) Heatmaps of CDX2 ChIP-Seq in Caco-2 cells with control or LIN28B KD. (**C**) Left: peak analysis annotated by promoter transcription starting sites; gray columns indicate the number of significant higher peaks compared with the other phenotype. Right: the gene list of peak annotation analysis. (**D**) Significant upregulated KEGG pathway analysis related to metabolism in Caco2 control cells compared with Caco2 LIN28B KD cells. (**E**) Significant upregulated gene ontology term analysis related to fatty acid metabolism in Caco2 control cells compared with Caco2 LIN28B KD cells. (**F**) CDX2 ChIP-Seq tag counts at the site of AMACR TSS promoter. The binomial test was performed with *P* < 0.05 as statistically significant (**A** and **C**–**E**). CDX2 binding peaks were identified by applying FDR cutoff 0.05.

We next hypothesized that CDX2 regulates downstream genes in the context of LIN28B overexpression. To evaluate this possibility, we performed ChIP with a CDX2 antibody followed by ChIP-Seq in Caco-2 control cells (endogenous high Lin28B expression) and Lin28B knockdown Caco-2 cells (Figure 5A). We especially focused on transcription starting sites (TSSs) in our analysis. We observed that CDX2 bound more TSSs and transcription ending sites (TESs) in parental Caco-2 cells than in the Lin28B knockdown Caco-2 cells (Figure 5B and Supplemental Figure 6, A and B). This suggests CDX2-mediated transcriptional activity might be enhanced in the context of LIN28B overexpression. Additionally, when we compared the peak level of CDX2 binding sites between these 2 cell lines, we found 667 sites had significantly higher peaks in Caco-2 control cells compared with LIN28B knockdown Caco-2 cells, whereas only 92 sites in LIN28B knockdown Caco-2 had higher peaks (Figure 5C). These results imply that LIN28B expression affected the affinity of CDX2 with the promoters of certain genes. From these results, we focused on AMACR, which is in the top 3 genes in peak annotation analysis (Figure 5C); some studies have reported that AMACR is expressed preferentially in differentiated CRCs (46–48). Several studies also suggest that AMACR expression may be a marker of tumor differentiation in prostate cancer (49) and breast cancer (50).

AMACR is a mitochondrial and peroxisomal enzyme that plays a critical role in peroxisomal beta-oxidation of branched-chain fatty acids (26). Indeed, Kyoto Encyclopedia of Genes and Genomes (KEGG) pathway analysis of our ChIP-Seq revealed that CDX2 bound to genes involved in metabolism, including fatty acid metabolism in Caco2 control cells (Figure 5D). Moreover, gene ontology (GO) terms revealed that genes associated with fatty acid metabolism were occupied highly by CDX2 in Caco2 control cells (Figure 5E). These results indicate that CDX2 might be involved in fatty acid metabolism in the context of LIN28B overexpression, and it is possible that AMACR may play an important role in this context. As shown in Figure 5F, the CDX2-bound region in the AMACR promoter-TSS in Caco2 control cells is significantly higher than that in Caco2 shLIN28B cells. This suggests that CDX2 may regulate AMACR transcriptionally.

**Figure 6:**
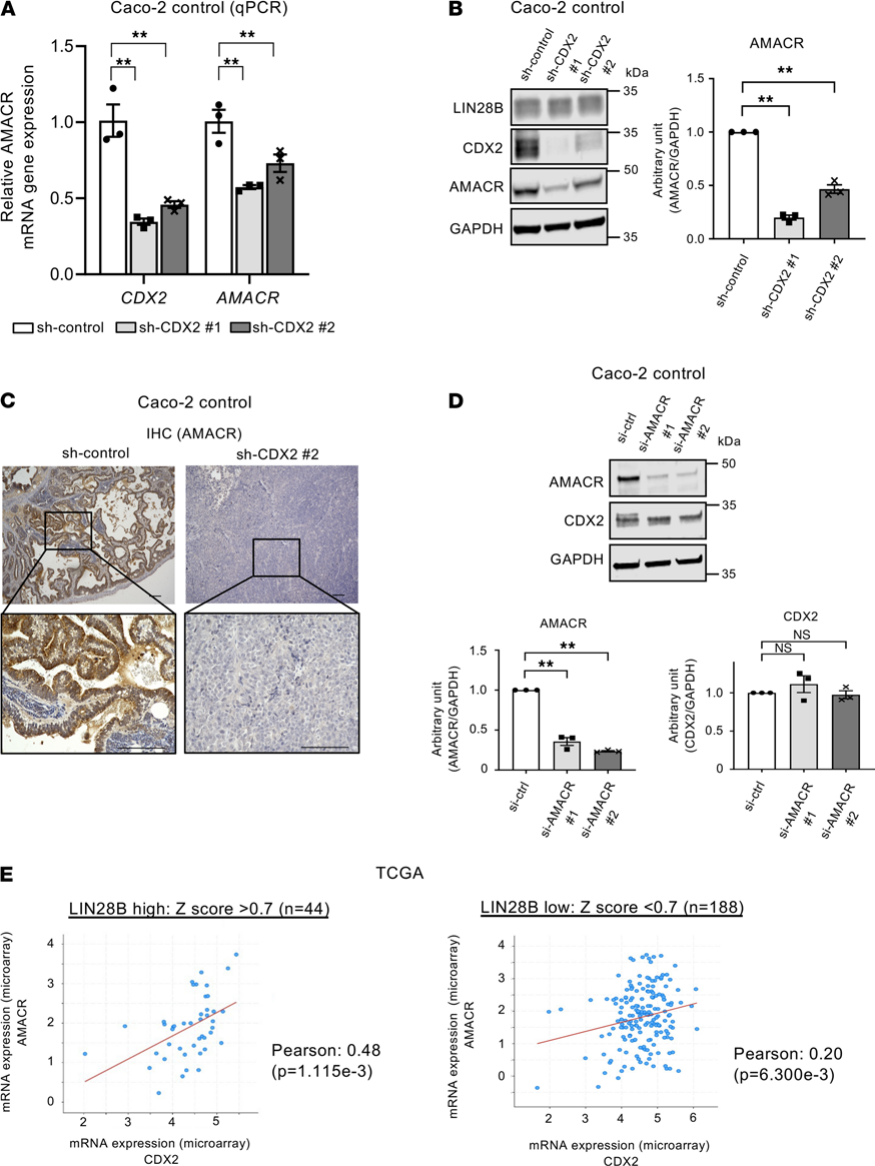
CDX2 expression has a positive correlation with AMACR expression in CRC in the context of LIN28B overexpression. (**A**) *CDX2* and *AMACR* expression (qPCR) in Caco-2 control/CDX2 KD cells (*n* = 3). (**B**) Left: WB analysis of CDX2 and AMACR in Caco-2 control and CDX2 KD cells. Right: band intensities were normalized by densitometry to GAPDH (*n* = 3). (**C**) Representative IHC staining for AMACR in the subcutaneous xenograft tumors of Caco-2 cells with control or CDX2 KD. Scale bars: 100 μm. (**D**) Left: WB analysis of CDX2 and AMACR in Caco-2 control and AMACR KD cells. Right: band intensities were normalized by densitometry to GAPDH (*n* = 3). (**E**) Correlation graph between mRNA expression of CDX2 and of AMACR in colon adenocarcinomas and rectal adenocarcinomas (COADREAD) datasets in The Cancer Genome Atlas (TCGA) in LIN28B high expression (upper) or LIN28B low expression (lower). Correlation of expression was determined via Pearson correlation coefficient test. Data are presented as mean ± SEM. One-way ANOVA followed by Dunnett’s multiple-comparison test as post hoc analysis (**A**, **B**, and **D**) or Pearson correlation coefficient test (**E**) was performed. *P <0.05, **P < 0.01.

We next investigated the potential functional relationship between CDX2 and AMACR. As shown in Figure 6, A and B, AMACR mRNA and protein expression were decreased in Caco-2 cells upon CDX2 knockdown. IHC staining revealed AMACR expression was decreased dramatically in subcutaneous xenograft tumors with CDX2 knockdown (Figure 6C). These results were also observed in constitutively expressing LIN28B LoVo cells (Supplemental Figure 7, A–C). We next generated Caco-2 WT (with high endogenous LIN28B expression) cells with siRNA-mediated AMACR knockdown. As shown in Figure 6D, AMACR knockdown did not affect CDX2 expression, consistent with it being downstream of CDX2.To validate the relationship between CDX2 and AMACR in human CRCs, we next evaluated The Cancer Genome Atlas (TCGA) for CDX2 and AMACR in colon and rectal adenocarcinomas (COADREAD). As shown in Figure 6E, we found a positive correlation between these 2 genes in LIN28B high-expressing CRCs (Pearson’s coefficient = 0.48, *P* < 0.01). By contrast, there was no correlation in LIN28B low-expressing CRCs (Pearson’s coefficient = 0.20, *P* < 0.01). Interestingly, we obtained the same trends from a human CRC tissue microarray (TMA) (Tables 3 and 4). In addition, we did not find a correlation between CDX2 and AMACR in LIN28B knockdown Caco2 cells (Supplemental Figure 7D) and LoVo empty vector cells, the latter of which had very low endogenous LIN28B expression (Supplemental Figure 7E). These results imply that CDX2-mediated regulation of AMACR may occur specifically in the context of only LIN28B overexpression in CRC.

### AMACR may contribute to the promotion of CRC cell differentiation in the context of high LIN28B expression.

**Figure 7:**
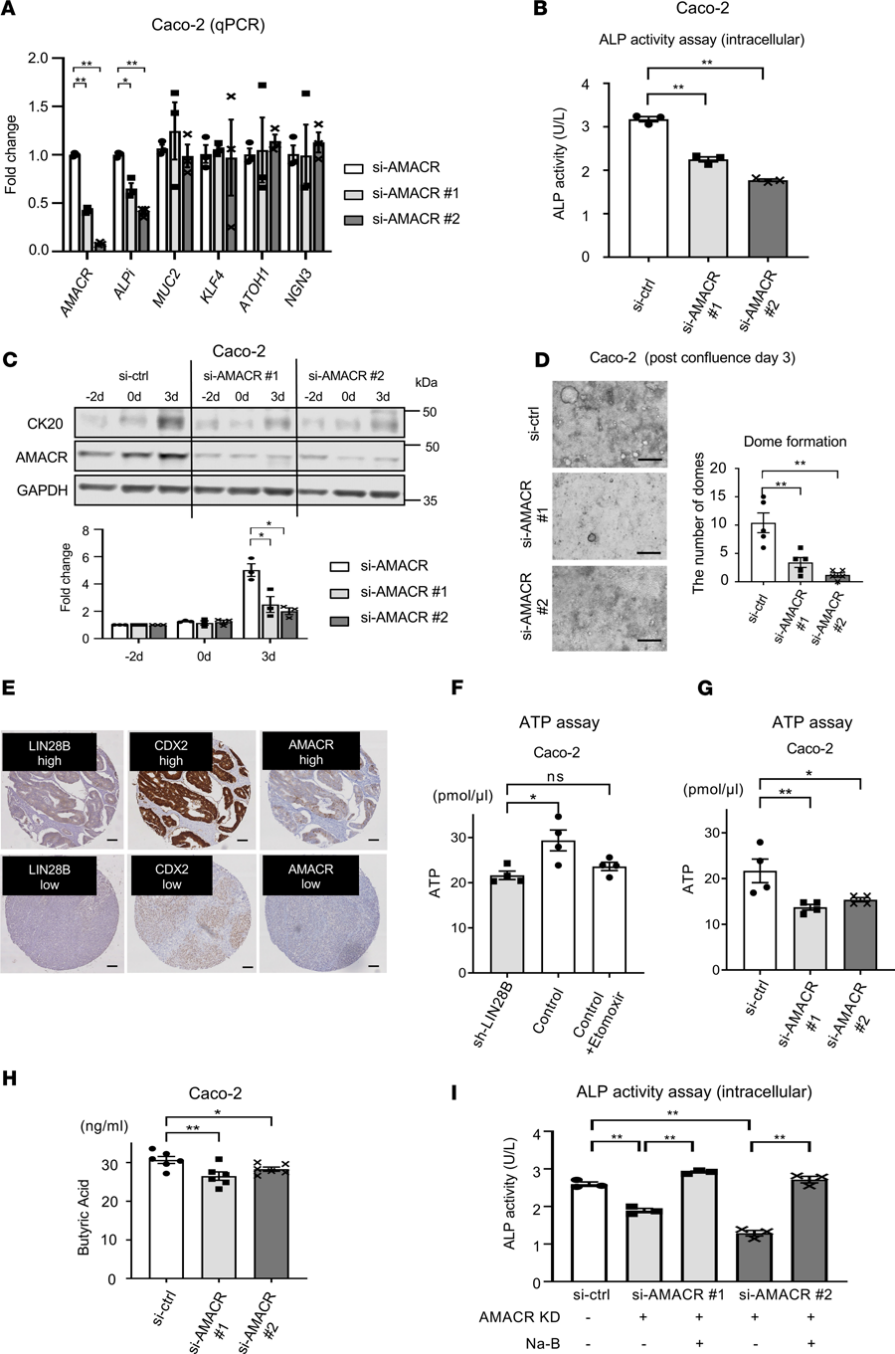
AMACR promotes CRC cell differentiation in the context of LIN28B overexpression. (**A**) qPCR for intestinal differentiation markers in Caco-2 control/AMACR KD Caco-2 cells (*n* = 3). (**B**) ALP activity assay in Caco-2 control/AMACR KD cells (*n* = 3). (**C**) Upper: WB analysis of AMACR and CK20 expression in Caco-2 cells with control or si-AMACR at the confluence time point. Lower: densitometry; the value for CK20 or CDX2 at –2 days for the sh-control samples was designated as 1. (**D**) Left: representative image for dome formation in Caco-2 cell at postconfluence day 3. Scale bars: 500 μm. Right: The graph indicates the number of domes. A dome was defined as greater than 100 μm diameter. (*n* = 5) (**E**) Representative IHC staining images of human CRC TMAs. (**F**) The ATP assay in Caco-2 with control and LIN28B KD cells (*n* = 4). (**G**) The ATP assay in Caco-2 with control and AMACR KD cells (*n* = 4). (**H**) Butyric acid measurement in Caco-2 with control and AMACR KD cells (*n* = 6). (**I**) ALP activity assay in Caco-2 control/AMACR KD cells with or without 1 mM sodium butyrate (Na-B) (*n* = 3). Data are presented as mean ± SEM. Unpaired, 2-tailed Student’s *t* test (**F**) and 1-way ANOVA followed by Dunnett’s multiple-comparison test as post hoc analysis (**A**–**D** and **G–I**) were performed. *P < 0.05, **P < 0.01.

We next executed in vitro studies with AMACR knockdown Caco-2 cells mediated by siRNA to investigate the function of AMACR in CRC cell differentiation. First, we evaluated several intestinal differentiation markers by qPCR in siRNA-mediated AMACR knockdown cells. Interestingly, we observed that AMACR regulated ALPi gene expression, whereas it did not affect gene expression for other intestinal cell (secretory) lineages, such as goblet cells (MUC2, KLF4) and enteroendocrine cells (ATOH1, NGN3) (Figure 7A). Furthermore, we found that intracellular ALP activity decreased significantly with AMACR knockdown in Caco-2 cells (Figure 7B). Next, to determine the effect of AMACR knockdown in CRC cell differentiation, we transiently transfected subconfluent Caco-2 cells with siAMACR and harvested cells at subconfluence (–2 days), confluence (day 0), and after confluence (day 3). As shown in Figure 7C, we observed that siRNA-mediated AMACR knockdown inhibited CK20 expression at the postconfluence time point. We found that dome formation in Caco-2 cells with siAMACR was reduced significantly compared with Caco-2 control cells (Figure 7D) at 3 days after confluence. We next generated AMACR-overexpressing LoVo cells (Supplemental Figure 8A). AMACR overexpression resulted in the upregulation of ALP activity in CRC cells (Supplemental Figure 8B). We also found that differentiated xenograft tumors with LIN28B had high ALP activity (Figure 3H). Equally important, most human CRCs that had concordant high LIN28B, CDX2, and AMACR expression (11/12; 91.6%) exhibited well to moderate differentiation status, whereas 2 of 3 CRCs with low LIN28B, CDX2, and AMACR expression exhibited poor differentiation status (Table 5 and Figure 7E).

Although some studies have revealed that AMACR expression may be a marker of tumor differentiation in several cancer types (46–48), the mechanism underlying AMACR-mediated tumor differentiation is still elusive. A number of studies have reported that butyric acid, a straight short-chain fatty acid, plays a crucial role in intestinal differentiation (51) and stimulates ALP activity (52, 53). It is also widely accepted that a metabolic hallmark of cancer cells involves lipidomic remodeling, which encompasses alterations in fatty acid transport, de novo lipogenesis to support membrane synthesis, and beta-oxidation to generate ATP (54). Given that AMACR is an enzyme involved in oxidation of branched-chain fatty acids, we hypothesized that AMACR may alter the level of butyric acid in CRC. To test this hypothesis, we first assessed ATP levels in Caco-2 cells with and without LIN28B knockdown. As shown in Figure 7F, LIN28B knockdown attenuated the ATP level in Caco-2 cells. In correlation, etomoxir, which is a known inhibitor of beta-oxidation, suppressed the enhancement of ATP seen with LIN28B overexpression. Furthermore, AMACR knockdown decreased the ATP level in Caco-2 cells (Figure 7G). We also found that the ATP level was diminished by CDX2 knockdown in this cell line (Supplemental Figure 8C), thereby suggesting the CDX2/AMACR axis may facilitate beta-oxidation in LIN28B-overexpressing CRC cells, resulting in increased intracellular energy levels. Next, we measured the level of short fatty acids in CRC cells with AMACR knockdown. As shown in Figure 7H, the intracellular butyric acid levels were significantly decreased in Caco-2 cells with AMACR knockdown compared with control cells. Moreover, the reduction of ALP activity in AMACR knockdown CRC cells was rescued by sodium butyrate addition in the cultured media (Figure 7I). The addition of sodium butyrate in LIN28B or CDX2 knockdown cells also resulted in enhanced ALP activity (Supplemental Figure 8, D and E). Taken together, these findings strongly suggest that the AMACR axis may promote CRC cell differentiation through butyric acid–mediated ALP activation in the context of LIN28B and CDX2 coexpression.

## Discussion

LIN28B and its homolog LIN28A function through the posttranscriptional downregulation of the *let-7* miRNA family. However, some LIN28B-mediated functions are also *let-7* independent through direct binding of specific mRNAs, such as *OCT4* (36), *BMP4* (55), and *NRP-1* (56) and putative targets based upon in silico analysis. In our previous work, cross-linking, IP, and high-throughput sequence (CLIP-Seq) analysis revealed a large number of putative mRNA targets of LIN28B in the small intestinal epithelium and CRC cells (8). In the present study, we provided the first demonstration, to our knowledge, that LIN28B upregulated CDX2 protein expression by directly binding to *CDX2* mRNA. Furthermore, in *Vil-LIN28B* mice, we observed LIN28B maintained CDX2 expression during malignant transformation. Conversely, 3 other studies demonstrated that CDX2 expression is reduced in tumors (37–39). To reconcile these apparent differences, it is possible that LIN28B overexpression promotes CDX2 maintenance and tumor differentiation, but in the absence of LIN28B overexpression, CDX2 expression is downregulated.

Although some studies have demonstrated that the absence of CDX2 in CRC correlates with poor prognosis (20, 25), only fewer than 10% of human CRCs lack CDX2 expression (24). Differentiated CRC tumors, which are strongly associated with CDX2 high expression, still have high metastatic potential (22). Furthermore, CDX2 expression status is highly concordant between primary CRCs and their corresponding liver metastases (22). This implies the preponderance of CRCs maintain CDX2 expression during metastatic progression. In this study, we demonstrated the mechanism for how the metastatic progression of differentiated CRC tumors occurs under the conditions of LIN28B overexpression leading to induction of CDX2.

In CRC, mortality is related to metastasis to distant organs, and further understanding of these mechanisms is critical to improve patient survival. Recent studies have provided concrete experimental data to support the notion that epithelial plasticity is essential for metastatic colonization (42, 43, 57). Since colonization requires tumor cells to restart proliferation upon extravasation into a metastatic site, MET may be required to support growth of metastatic foci after initial seeding. Indeed, we demonstrated CDX2 facilitates CRC cell proliferation at the metastatic site through the upregulation of E-cadherin, which is needed for MET. E-cadherin is an intercellular adhesion protein fulfilling a prominent role in epithelial differentiation, which implies CDX2 may play a role in CRC metastatic progression while maintaining the tumor’s differentiation status through the induction of E-cadherin.

CDX2 is well characterized as a CRC differentiation marker, and several lines of evidence indicate that CDX2 loss is associated with poorly differentiated CRC (20, 25). Herein, we demonstrated that LIN28B overexpression enhanced CDX2 expression to facilitate CRC differentiation. Given that LIN28B is known to be a key contributor to the formation of induced pluripotent stem (iPS) cells (58), it is possible that LIN28B is reprograming CRC cells to a more differentiated identity and fate through CDX2. Interestingly, a recent study revealed CDX2 is indispensable for differentiation from iPS cells (59).

We have identified AMACR as a potentially novel target gene in the LIN28B/CDX2 axis through ChIP-Seq with a CDX2 antibody in Caco2-cells. AMACR has been used as an important diagnostic marker to distinguish normal glands from cancer in the prostate (60). Intriguingly, several studies have demonstrated that AMACR is expressed preferentially in differentiated CRCs and its precursor lesions (adenomas) (46–48, 61). Some studies have speculated that AMACR overexpression may lead to alterations in the balance of cellular oxidants through its role in fatty acid metabolism, which in turn may contribute to the pathogenesis of neoplasia (62, 63). The mechanism of how AMACR mediates CRC differentiation (or any other type of differentiated cancer), however, remained unknown until now. Our ChIP-Seq revealed that pathways and GO terms associated with fatty acid metabolism were enriched in parental Caco2 cells compared with Caco-2 shLIN28B cells. Indeed, we demonstrated LIN28B and subsequent upregulated AMACR expression promoted beta-oxidation and ATP production in CRC cells. Some studies also indicate that butyric acid, which is a short-chain fatty acid known to be an inducer of intestinal differentiation (51), stimulates ALP activity (52, 53). ALP activity is used to evaluate differentiation toward the colonocyte lineage in CRC cells (34). Our study revealed that AMACR might enhance ALPi activity through the upregulation of butyric acid in LIN28B-overexpressing CRC cells. Further investigation will be warranted to elucidate how AMACR upregulates butyric acid, made challenging because of the complexity of altered fatty acid metabolism in cancer (64) and the role of native colonic microbiota in fatty acid production and breakdown. However, given that AMACR is an enzyme involved in oxidation of branched-chain fatty acids, it is possible that enhanced AMACR by LIN28B may induce a shift in the intracellular lipid profile in order to use branched chain fatty acids as primary sources for ATP to allow butyrate to serve other critical roles in tumor promotion, including colonocyte differentiation via ALP. Butyrate has several additional roles in immune modulation, signaling via G protein–coupled receptors, and histone deacetylase inhibition (65, 66), all of which may have a role in CRC progression.

In conclusion, we demonstrated a mechanism for CRC differentiation through a potentially novel LIN28B high/CDX2/AMACR axis, thus elucidating how CRC differentiation and CRC metastatic progression are regulated at the molecular level.

## Methods

### Cell lines.

Human CRC cell lines (Caco-2, LoVo, DLD1, RKO, HCT-116, and SW480) were obtained from American Type Culture Collection. These cell lines were authenticated by the STR locus and were maintained in DMEM (Thermo Fisher Scientific), supplemented with 10% FBS (GE Healthcare Life Sciences), and 1% penicillin-streptomycin (Thermo Fisher Scientific) in a 37 °C incubator with 5% CO_2_. Cells were tested for mycoplasma at least every 2 months.

### Transfection.

LIN28B knockdown in Caco-2 cells and LIN28B long-isoform overexpression (rescue) in LIN28B knockdown Caco2 cells was performed as described previously (28). CDX2 knockdown in Caco-2 cells and LoVo cells was performed using Genecopoeia shRNA system with HSH000553-31-LVRU6MH (shCDX2 no. 1) and HSH000553-33 LVRU6MH (shCDX no. 2) and CSHCTR001-LVRU6MH (sh-control) constructs. AMACR overexpression in Caco-2 cells and LoVo cells was performed using Genecopoeia overexpression system with EX-Z1680-Lv151 (AMACR o/e) and EX-NEG-Lv151 (empty vector) constructs. Next, 6 × 10^5^ Caco2 or LoVo cells were seeded in 6-well plates and 16 hours later infected by applying virus-containing media plus polybrene (4 μg/mL) to cells and then subjecting them to centrifugation at 1800 *g* for 90 minutes. These cells were selected with 200 or 100 μg/mL hygromycin (Sigma-Aldrich), respectively.

### RNAi transfection.

Cells were transfected using Lipofectamine RNAiMAX (Thermo Fisher Scientific) and a final concentration of 10 nM siRNA in Opti-MEM I reduced serum medium (Thermo Fisher Scientific). AMACR siRNA were purchased from Sigma-Aldrich; control; SIC001, si-AMACRno. 1; SASI_Hs01_00189298, si-AMACR no. 2; SASI_Hs01_00189299.

### Quantitative real-time PCR.

Total RNA from cells was isolated with the GeneJet RNA purification kit (Thermo Fisher Scientific). RT reactions were performed with the High-Capacity Reverse Transcription Kit with RNase inhibitor (Applied Biosystems), according to the manufacturer’s instructions. qPCR utilized the Fast SYBR (Invitrogen) master mixes. The expression levels of mRNA were normalized to GAPDH. Primer sequences are listed in Supplemental Table 1. Gene expression data are expressed as a fold-change normalized to the mean values for controls. All experiments were conducted in at least 3 independent settings with technical replicates (duplicates) in each experiment.

### Western blotting.

Proteins from cells or tissues were isolated using NP40 lysis buffer, and Western blots were performed with the Novex NuPAGE SDS-PAGE gel system (Invitrogen) in MOPS-SDS, according to the manufacturer’s instructions as described previously (8). Proteins were visualized with an Odyssey Infrared Imager (LI-COR Biosciences) for near-infrared (near-IR) fluorophore-conjugated antibodies. Near-IR fluorescence was quantified using LI-COR Image Studio Software. Primary antibodies used for Western blot analysis are listed in Supplemental Table 2.

### RIP assay.

RNA IP assays were performed using the RiboCluster Profiler RIP-Assay Kit (RN1005, MBL), according to the manufacturer’s instructions. Briefly, 1 × 10^7^ LIN28B-overexpressing Caco-2 or LoVo cells were lysed in the provided buffer supplemented with 1.5 mM dithiothreitol (Invitrogen), 100 units/mL RNase inhibitor (RNaseOUT, Invitrogen), and protease inhibitors. Cell lysates were precleared using 100 μL of SureBeads Protein G Magnetic Beads (Bio-Rad Laboratories) for 1 hour at 4°C. Precleared cell lysates were incubated at 4°C for 3 hours with Protein G Magnetic Beads that were preincubated with anti-Lin28B Ab and control rabbit IgG (provided in the kit). A magnet was used to capture the antibody-immobilized beads and the supernatant was discarded. Beads were then washed with the provided washing buffer supplemented with 1.5 mM of DTT. In the last wash, the part of washing buffer was removed for Western blot analysis. RNA isolation was performed according to the manufacturer’s protocol. RNA was subjected to RT-PCR analyses using primers for the cDNAs of CDX2, OCT4, and SOX2.

### ALP activity assay.

ALP activity assays were performed using Alkaline Phosphatase Activity Colorimetric Assay Kit (K412-500, BioVision) according to the manufacturer’s instructions. Briefly, washed 2 × 10^5^ Caco-2 cells were homogenized in ALP assay buffer, and then 80 μL of supernatant after centrifuging was put into a 96-well plate. We then added 50 μL of the 5 mM p-nitrophenyl phosphate solution as a phosphatase substrate, which turns yellow (λ_max_ = 405 nm) when dephosphorylated by ALP. After incubation of the reaction for 60 minutes at 25°C, all reactions were stopped by adding 20 μL stop solution and optical density measured at 405 nm in a microplate reader (67). ALP activity was also determined immunohistochemically using a red alkaline phosphatase substrate kit I (Vector Laboratories) according to the manufacturer’s instructions. Briefly, after tissue sections were deparaffinized, they were incubated with the Vector red ALP substrate working solution at 4°C overnight.

### mRNA stability assay.

LIN28B-overexpressing Caco-2 and LoVo cells and control cells were incubated with actinomycin D (Caco-2, 15 µg/mL; LoVo, 3 µg/mL) for the indicated time. Total RNA from cells was isolated and qPCR was performed as described above.

### Transwell invasion assay.

First, 8 μm membrane pores coated with Matrigel (Corning) for 24-well plates were used according to the manufacturer’s guidelines. Briefly, chambers were rehydrated in serum-free DMEM for 2 hours at 37°C; 5.0 × 10^4^ CRC cells were seeded in the upper chamber in 100 μL serum-free DMEM, and DMEM with 10% FBS was added to the bottom of the well. Cells were incubated for 24 hours at 37°C. After incubation, noninvading cells were removed from the upper surface with a cotton swab, and the remaining cells were fixed in 100% methanol at –20°C for 15 minutes and stained by 0.2% crystal violet. The number of migratory or invasive cells was counted in 3 different fields by Keyence BZ-X810.

### Dome formation assay.

Caco-2 cells were cultured on 6-well plate at 3 days after confluency. The number of domes, which was defined as more than 100 μm diameter, was counted in 3 different fields.

### ATP assay.

ATP assays were performed using ATP Assay Kit (MAK190, Sigma-Aldrich), according to the manufacturer’s instructions. Briefly, washed 1 × 10^6^ Caco-2/LoVo cells were lysed in 100 μL of ATP assay buffer and deproteinized using a 10 kDa MWCO spin filter. Next, 80 μL of the samples was added into 50 μL of the reaction mix (ATP probe+ ATP converter+ developer mix) and incubated at room temperature for 30 minutes protected from light. ATP values were measured by the fluorescence (FLU, λex = 535/λem = 587 nm) in a microplate reader.

### Short fatty acid measurement.

siRNA-mediated AMACR knockdown/control Caco-2 cells in a 100 mm plate were washed gently with chilled PBS solution, and then the plates were scraped on ice with 750 μL of chilled HPLC grade 100% methanol.

The internal standards (^13^C_2_-d_3_, C_3_-d_5_, C_4_-d_7_, C_5_-d_9_, C_6_-d_11_) were added to the samples and derivatized with 3-nitrophenylhydrazine (3-NPH), and standard curves were performed with the samples for the quantification, analyzed using an AB Sciex 6500+ QTRAP MS, and ultra-performance liquid chromatography (UPLC). UPLC separation was performed with a Waters BEH C18 column, the data collected with the AB Sciex 6500+ QTRAP MS with positive MRM mode, and the data processed with AB Sciex MultiQuant software.

### Animal models.

We generated LIN28B-overexpressing transgenic animals as reported previously (8). Briefly, a FLAG-HA–tagged mouse LIN28B cDNA and an IRES-tdTomato expression cassette were cloned downstream of the 13 Kb mouse villin (*Vil1*) promoter (8) in the p13KVil-SVLpA vector. The transgene was linearized and removed from vector backbone sequences by digestion with PmeI (New England Biolabs). After purification, the *Vil-Lin28b* transgene was injected in B6SJL F1 fertilized oocytes.

### Xenograft experiments.

Eight-week old female CrTac:NCr-Foxn1nu nude athymic mice (Taconic) were irradiated with 5 Gy 2–3 hours prior to injection of cells. LIN28B-overexpressing Caco-2 and LoVo cells with or without lentiviral constructs (CDX2 sh-RNA no. 1, CDX2 sh-RNA no. 2, or sh-RNA Scr) were trypsinized. Cells were resuspended at a concentration of 8 x 10^7^ cells/mL and 4 x 10^7^ cells/mL with DMEM, respectively. Next, 50 μL of cell suspensions were mixed with 50 μL of Matrigel Basement Membrane Matrix (BD Biosciences) to achieve a volume of 100 μL containing 4 × 10^6^ Caco-2 cells or 2 × 10^6^ LoVo cells per injection. Cells were s.c. injected into the rear flanks of mice sedated with ketamine (100 mg/kg) and xylazine (10 mg/kg). After injection, mice were monitored periodically. Tumor diameters were measured with digital calipers, and the tumor volume in mm^3^ was calculated by the formula: volume = (width)^2^ × length/2. Mice were euthanized after 4 weeks. All mice were cared for in accordance with University Laboratory Animal Resources requirements.

### Portal vein injection.

Female nude mice at the age of 9 weeks were injected with 2 × 10^6^ CRC cells infected with lentivirus into the portal vein under anesthesia. After 28 days, the mice were euthanized and the whole liver was removed. Liver metastases were counted as previously described (68).

### Histology, IHC, and tissue analyses.

Tissues were fixed in zinc formalin fixative overnight at 4°C, washed in PBS, and moved to 70% ethanol before paraffin embedding and sectioning. H&E staining was performed according to standard procedure (69). For immunostaining, antigen retrieval was performed by heating slides in 10 mM citric acid buffer (2.1 g citric acid monohydrate in 1 L di H_2_O, pH 6.0) in a pressure cooker. The images were taken using Keyence BZ-x800. Imaging was performed at RT. Primary antibodies used for IHC analysis are listed in Supplemental Table 2. The number of Ki-67–positive cells was counted by Keyence BZ-X810.

### Human CRC specimens.

The human colon cancer TMAs were obtained from deidentified patients who underwent surgery for colon cancer at Columbia University, which was exempt from the IRB. Formalin-fixed and paraffin-embedded samples were stained with the antibodies in Supplemental Table 2 using standard IHC techniques. The staining intensities of expression and tumor differentiation status were evaluated by 2 investigators. The staining intensities varied from 0 (negative), 1+ (weak), 2+ (moderate), to 3+ (strong). The percentage of cells at each staining intensity level was calculated, and H-score was assigned using the following formula: H-score = 1 × (% cells 1+) + 2 × (% cells 2+) + 3 × (% cells 3+). High expression levels were designated as follows: LIN28B 120 or higher, CDX2 200 or higher, AMACR 100 or higher.

### TCGA analysis.

Publicly available gene expression data from TCGA were downloaded from cBio portal (70, 71) and graphs were generated using the same site. For Lin28B expression, expression groups were determined by *z* score (*z* score > 0.7, high expression; *z* score > 0.3, positive expression). Correlation of expression was determined via Pearson correlation coefficient test.

### ChIP, ChIP-Seq, and data analysis.

First, 2 × 10^7^ Caco-2 control and LIN28B knockdown cells were cross-linked with 1% formaldehyde for 10 minutes at 37°C, washed in cold PBS, resuspended in lysis buffer (1% SDS, 10 mM EDTA, 50 mM Tris-HCL, pH 8.1, and complete protease inhibitors; Roche), and sonicated to obtain chromatin fragments between 200 bp and 1000 bp. Sonicated chromatin was resuspended in IP buffer (1% Triton X-100, 2 nM EDTA, 150 mM NaCl, 20 mM Tris-HCl, pH 8.1) and incubated overnight at 4°C with magnetic beads conjugated to CDX2 (Bethyl, BL3194). The IP was washed 5 times with RIPA buffer (50 mM HEPES, pH 7.6, 1 MM EDTA, 0.7% Na deoxycholate, 1% NP-40, 0.5 M LiCl) and the DNA recovered by reversing the cross-link in 1% SDS, 0.1 M NaHCO_3_ for 8 hours at 65°C. DNA was purified and quantified by Qubit. For ChIP-Seq, 30 ng each of ChIP were processed for deep sequencing by Genewiz. Prior to sequencing, qPCR was used to verify that positive and negative control ChIP regions were amplified in the linear range. IP chromatin of Caco2 control cells was normalized to IP chromatin of Caco2 LIN28B knockdown cells.

FASTQ files from Illumina sequencer were trimmed by Trimmomatic 0.32 (72) to remove low-quality bases. The trimmed reads were mapped to mm10 mouse reference genome using bowtie2 version 2.3.4.1 (73). The mapped reads were filtered by MAPQ >10 and sorted, and the duplicated reads were removed afterward for downstream analysis. The Bigwig files were generated using HOMER (74) function to make a UCSC file in which the total counts were normalized to 10 × 10^7^ per sample. The MACS2 (75) algorithm was used to call peaks. CDX2 binding peaks were identified by applying FDR cutoff 0.05.

### Data availability.

The dataset produced in this study (ChIP-Seq data) is available in the NCBI’s Gene Expression Omnibus (GEO GSE165674).

### Statistics.

One-way ANOVA followed by Dunnett’s multiple-comparison test analyzed differences between endpoint measurements across all 3 experimental groups. Unpaired, 2-tailed Student’s *t* tests were performed to determine statistical significance of comparisons between 2 groups. Fisher’s exact tests were used to compare the distribution of a categorical variable in a group with the distribution in another group; *P* less than 0.05 was considered statistically significant. Statistical analysis was performed using GraphPad Prism software version 8.0. For all analyses, data from a minimum of 3 independent experiments are presented as mean ± SEM. The sample size for each experiment is included in the figure legends.

### Study approval.

All animal studies were approved by the IACUC at the University of Pennsylvania and Columbia University.

## Author contributions

KS, RM, and AKR conceived the study. KS, RM, YM, UMS, and AKR performed the methodology. KS, RM, YM, PC, and SFA performed the investigation. SK performed bioinformatics and ChIP data analysis. AJKS, SB, and HER performed the histological analysis. KS and AKR wrote the original draft of the manuscript. KS, YM, UMS, PC, MPV, and AKR reviewed and edited the manuscript.

## Supplementary Material

Supplemental data

## Figures and Tables

**Table 1:**
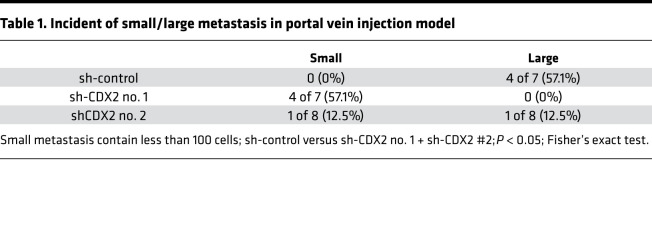
Incident of small/large metastasis in portal vein injection model

**Table 2:**
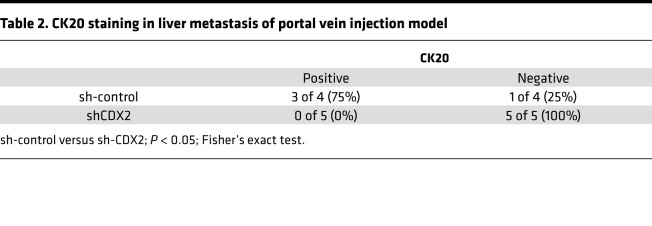
CK20 staining in liver metastasis of portal vein injection model

**Table 3:**
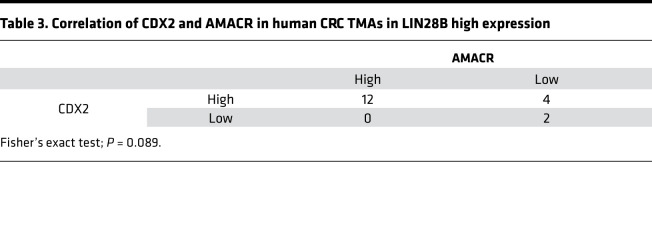
Correlation of CDX2 and AMACR in human CRC TMAs in LIN28B high expression

**Table 4:**
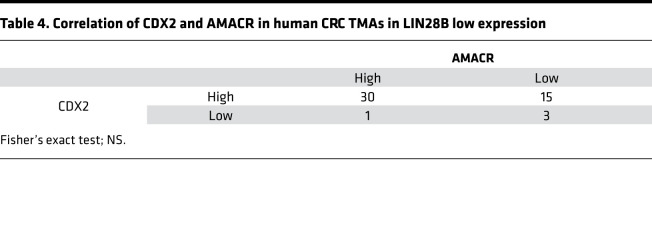
Correlation of CDX2 and AMACR in human CRC TMAs in LIN28B low expression

**Table 5:**
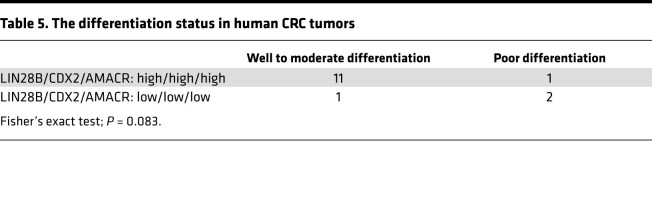
The differentiation status in human CRC tumors

## Version 1 03/23/2021

In-Press Preview

## Version 2 05/10/2021

Electronic publication

## References

[1] Muller-McNicoll M, Neugebauer KM (2013). How cells get the message: dynamic assembly and function of mRNA-protein complexes. Nat Rev Genet.

[2] Hentze MW (2018). A brave new world of RNA-binding proteins. Nat Rev Mol Cell Biol.

[3] Balzeau J (2017). The LIN28/let-7 pathway in cancer. Front Genet.

[4] Chatterji P, Rustgi AK (2018). RNA binding proteins in intestinal epithelial biology and colorectal cancer. Trends Mol Med.

[5] Masuda K, Kuwano Y (2019). Diverse roles of RNA-binding proteins in cancer traits and their implications in gastrointestinal cancers. Wiley Interdiscip Rev RNA.

[6] Viswanathan SR (2009). Lin28 promotes transformation and is associated with advanced human malignancies. Nat Genet.

[7] King CE (2011). LIN28B promotes colon cancer progression and metastasis. Cancer Res.

[8] Madison BB (2013). LIN28B promotes growth and tumorigenesis of the intestinal epithelium via Let-7. Genes Dev.

[9] Madison BB (2015). Let-7 represses carcinogenesis and a stem cell phenotype in the intestine via regulation of Hmga2. PLoS Genet.

[10] Tu HC (2015). LIN28 cooperates with WNT signaling to drive invasive intestinal and colorectal adenocarcinoma in mice and humans. Genes Dev.

[11] Chatterji P (2018). The LIN28B-IMP1 post-transcriptional regulon has opposing effects on oncogenic signaling in the intestine. Genes Dev.

[12] Piskounova E (2011). Lin28A and Lin28B inhibit let-7 microRNA biogenesis by distinct mechanisms. Cell.

[13] Kawahara H (2011). Musashi1 cooperates in abnormal cell lineage protein 28 (Lin28)-mediated let-7 family microRNA biogenesis in early neural differentiation. J Biol Chem.

[14] Wilbert ML (2012). LIN28 binds messenger RNAs at GGAGA motifs and regulates splicing factor abundance. Mol Cell.

[15] Gao N (2009). Establishment of intestinal identity and epithelial-mesenchymal signaling by Cdx2. Dev Cell.

[16] Silberg DG (2000). Cdx1 and cdx2 expression during intestinal development. Gastroenterology.

[17] Verzi MP (2010). Differentiation-specific histone modifications reveal dynamic chromatin interactions and partners for the intestinal transcription factor CDX2. Dev Cell.

[18] Crissey MA (2011). Cdx2 levels modulate intestinal epithelium maturity and Paneth cell development. Gastroenterology.

[19] Dalerba P (2016). CDX2 as a prognostic biomarker in stage II and stage III colon cancer. N Engl J Med.

[20] Hinoi T (2001). Loss of CDX2 expression and microsatellite instability are prominent features of large cell minimally differentiated carcinomas of the colon. Am J Pathol.

[21] Gross I (2008). The intestine-specific homeobox gene Cdx2 decreases mobility and antagonizes dissemination of colon cancer cells. Oncogene.

[22] Shigematsu Y (2018). CDX2 expression is concordant between primary colorectal cancer lesions and corresponding liver metastases independent of chemotherapy: a single-center retrospective study in Japan. Oncotarget.

[23] Kuo IM (2015). Clinical features and prognosis in hepatectomy for colorectal cancer with centrally located liver metastasis. World J Surg Oncol.

[24] Augestad KM (2015). Metastatic spread pattern after curative colorectal cancer surgery. A retrospective, longitudinal analysis. Cancer Epidemiol.

[25] Dalerba P (2016). CDX2 as a prognostic biomarker in colon cancer. N Engl J Med.

[26] Lloyd MD (2013). α-Methylacyl-CoA racemase (AMACR): metabolic enzyme, drug metabolizer and cancer marker P504S. Prog Lipid Res.

[27] Vachon PH, Beaulieu JF (1992). Transient mosaic patterns of morphological and functional differentiation in the Caco-2 cell line. Gastroenterology.

[28] Mizuno R (2018). Differential regulation of *LET-7* by LIN28B isoform-specific functions. Mol Cancer Res.

[29] Kusano Y (2012). Constitutive expression of an antioxidant enzyme, glutathione S-transferase P1, during differentiation of human intestinal Caco-2 cells. Free Radic Biol Med.

[30] Pereira B (2013). CDX2 regulation by the RNA-binding protein MEX3A: impact on intestinal differentiation and stemness. Nucleic Acids Res.

[31] Dalerba P (2011). Single-cell dissection of transcriptional heterogeneity in human colon tumors. Nat Biotechnol.

[32] Kim JH (2013). Loss of CDX2/CK20 expression is associated with poorly differentiated carcinoma, the CpG island methylator phenotype, and adverse prognosis in microsatellite-unstable colorectal cancer. Am J Surg Pathol.

[33] Matsumoto H (1990). Biosynthesis of alkaline phosphatase during differentiation of the human colon cancer cell line Caco-2. Gastroenterology.

[34] Wang Y (2017). Self-renewing monolayer of primary colonic or rectal epithelial cells. Cell Mol Gastroenterol Hepatol.

[35] Polesskaya A (2007). Lin-28 binds IGF-2 mRNA and participates in skeletal myogenesis by increasing translation efficiency. Genes Dev.

[36] Qiu C (2010). Lin28-mediated post-transcriptional regulation of Oct4 expression in human embryonic stem cells. Nucleic Acids Res.

[37] Oshima M (1995). Loss of Apc heterozygosity and abnormal tissue building in nascent intestinal polyps in mice carrying a truncated Apc gene. Proc Natl Acad Sci U S A.

[38] Aoki K (2003). Colonic polyposis caused by mTOR-mediated chromosomal instability in Apc+/Delta716 Cdx2+/- compound mutant mice. Nat Genet.

[39] Saandi T (2013). Regulation of the tumor suppressor homeogene Cdx2 by HNF4α in intestinal cancer. Oncogene.

[40] Kakiuchi Y (2002). Cyclooxygenase-2 activity altered the cell-surface carbohydrate antigens on colon cancer cells and enhanced liver metastasis. Cancer Res.

[41] Wang X (2015). Investigation of the roles of exosomes in colorectal cancer liver metastasis. Oncol Rep.

[42] Reichert M (2018). Regulation of epithelial plasticity determines metastatic organotropism in pancreatic cancer. Dev Cell.

[43] Takano S (2016). Prrx1 isoform switching regulates pancreatic cancer invasion and metastatic colonization. Genes Dev.

[44] Ezaki T (2007). The homeodomain transcription factors Cdx1 and Cdx2 induce E-cadherin adhesion activity by reducing beta- and p120-catenin tyrosine phosphorylation. Am J Physiol Gastrointest Liver Physiol.

[45] Funakoshi S (2010). Intestine-specific transcription factor Cdx2 induces E-cadherin function by enhancing the trafficking of E-cadherin to the cell membrane. Am J Physiol Gastrointest Liver Physiol.

[46] Jiang Z (2003). A dietary enzyme: alpha-methylacyl-CoA racemase/P504S is overexpressed in colon carcinoma. Cancer Detect Prev.

[47] Lin A (2007). Differential expression of alpha-methylacyl-coenzyme A racemase in colorectal carcinoma bears clinical and pathologic significance. Hum Pathol.

[48] Shi X (2007). Alpha-methylacyl-CoA racemase/P504S overexpression in colorectal carcinoma is correlated with tumor differentiation. Appl Immunohistochem Mol Morphol.

[49] Kuefer R (2002). alpha-Methylacyl-CoA racemase: expression levels of this novel cancer biomarker depend on tumor differentiation. Am J Pathol.

[50] Witkiewicz AK (2005). Alpha-methylacyl-CoA racemase protein expression is associated with the degree of differentiation in breast cancer using quantitative image analysis. Cancer Epidemiol Biomarkers Prev.

[51] Kaiko GE (2016). The colonic crypt protects stem cells from microbiota-derived metabolites. Cell.

[52] Iwami K, Moriyama T (1993). Effects of short chain fatty acid, sodium butyrate, on osteoblastic cells and osteoclastic cells. Int J Biochem.

[53] Orchel A (2005). Butyrate-induced differentiation of colon cancer cells is PKC and JNK dependent. Dig Dis Sci.

[54] Monaco ME (2017). Fatty acid metabolism in breast cancer subtypes. Oncotarget.

[55] Ma W (2013). Lin28 regulates BMP4 and functions with Oct4 to affect ovarian tumor microenvironment. Cell Cycle.

[56] Wang X (2018). RNA binding protein Lin28B confers gastric cancer cells stemness via directly binding to NRP-1. Biomed Pharmacother.

[57] Tsai JH (2012). Spatiotemporal regulation of epithelial-mesenchymal transition is essential for squamous cell carcinoma metastasis. Cancer Cell.

[58] Yu J (2007). Induced pluripotent stem cell lines derived from human somatic cells. Science.

[59] Matsuno K (2016). Redefining definitive endoderm subtypes by robust induction of human induced pluripotent stem cells. Differentiation.

[60] Rubin MA (2002). alpha-Methylacyl coenzyme A racemase as a tissue biomarker for prostate cancer. JAMA.

[61] Shukla N (2017). Expression of Alpha — Methylacyl — Coenzyme A Racemase (AMACR) in colorectal neoplasia. J Clin Diagn Res.

[62] Luo J (2002). Alpha-methylacyl-CoA racemase: a new molecular marker for prostate cancer. Cancer Res.

[63] Zhou M (2002). Alpha-Methylacyl-CoA racemase: a novel tumor marker over-expressed in several human cancers and their precursor lesions. Am J Surg Pathol.

[64] Koundouros N, Poulogiannis G (2020). Reprogramming of fatty acid metabolism in cancer. Br J Cancer.

[65] Chang PV (2014). The microbial metabolite butyrate regulates intestinal macrophage function via histone deacetylase inhibition. Proc Natl Acad Sci U S A.

[66] Flint HJ (2012). The role of the gut microbiota in nutrition and health. Nat Rev Gastroenterol Hepatol.

[67] Holscher HD (2014). Human milk oligosaccharides influence maturation of human intestinal Caco-2Bbe and HT-29 cell lines. J Nutr.

[68] Suzuki K (2017). Metadherin promotes metastasis by supporting putative cancer stem cell properties and epithelial plasticity in pancreatic cancer. Oncotarget.

[69] Andres SF (2019). IMP1 3’ UTR shortening enhances metastatic burden in colorectal cancer. Carcinogenesis.

[70] Cerami E (2012). The cBio cancer genomics portal: an open platform for exploring multidimensional cancer genomics data. Cancer Discov.

[71] Gao J (2013). Integrative analysis of complex cancer genomics and clinical profiles using the cBioPortal. Sci Signal.

[72] Bolger AM (2014). Trimmomatic: a flexible trimmer for Illumina sequence data. Bioinformatics.

[73] Langmead B, Salzberg SL (2012). Fast gapped-read alignment with Bowtie 2. Nat Methods.

[74] Heinz S (2010). Simple combinations of lineage-determining transcription factors prime cis-regulatory elements required for macrophage and B cell identities. Mol Cell.

[75] Zhang Y (2008). Model-based analysis of ChIP-seq (MACS). Genome Biol.

