# Supplementary material for: LIN28B induces a differentiation program through CDX2 in colon cancer
Source: JCI Insight. 2021 May 10;6(9):e140382. doi: 10.1172/jci.insight.140382 (PMC8262288; doi:10.1172/jci.insight.140382)
Supplement: Supplemental data [file jciinsight-6-140382-s223.pdf]

Supplematal Figures and legends

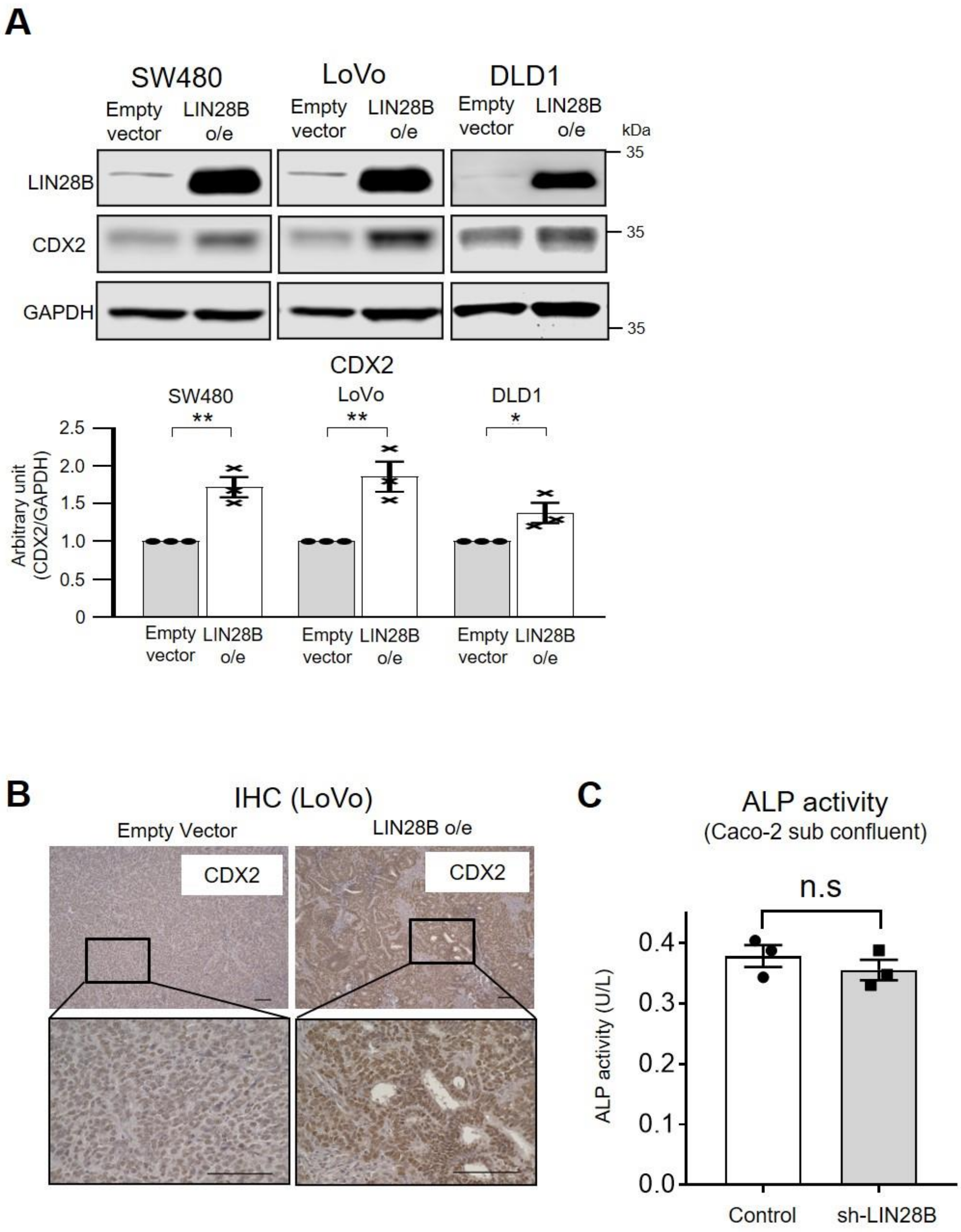

Supplemental Figure 1. LIN28B upregulates CDX2 expression in colorectal cancer cell lines. (A) Upper: WB analysis of LIN28B and CDX2 in SW480/LoVo/DLD1 control (Empty Vector) and LIN28B overexpression (o/e) cells. **Lower:** Band intensities were normalized by densitometry to GAPDH. (n=3) **(B)** Representative IHC staining for CDX2 in the subcutaneous xenograft tumor of LoVo cells with control or LIN28B KD. Scale bars, 100µm. **(C)** ALP activity assay in sub confluent Caco-2 cells. (n=3) Data are presented as mean ± SEM. Unpaired, two-tailed student's t-test was performed. (*) $p < 0.05$, (**) $p<0.01$.

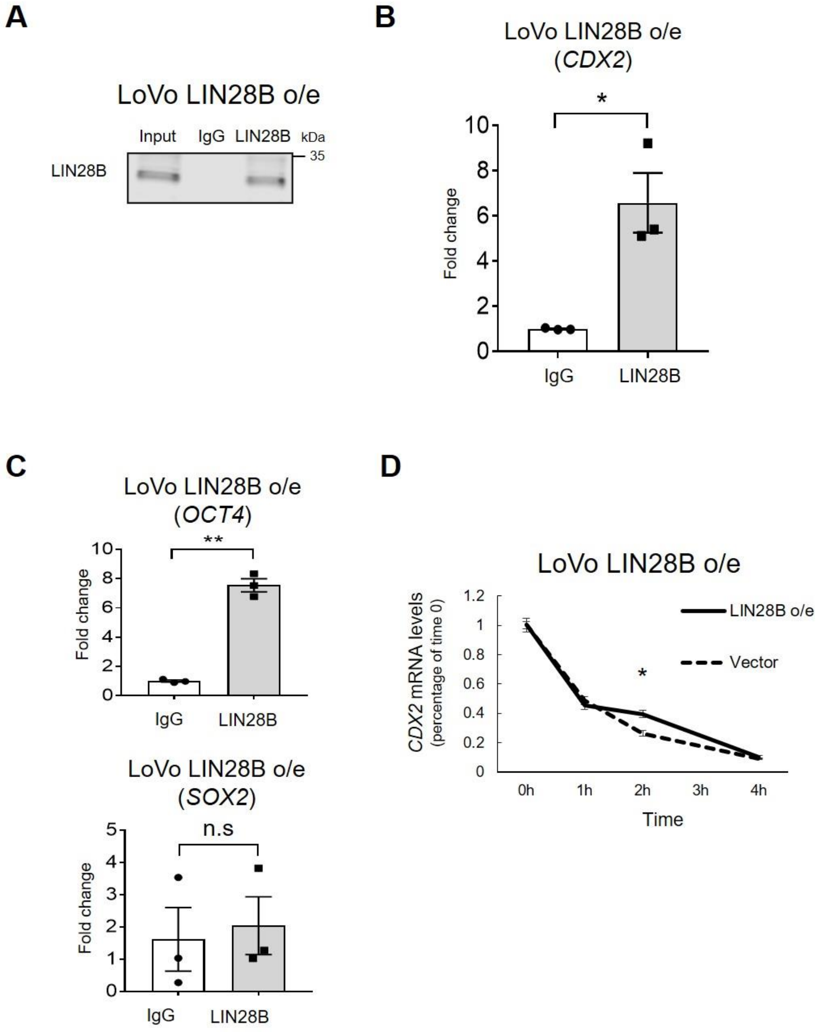

Supplemental Figure 2. LIN28B stabilizes CDX2 mRNA through direct binding. (A) Quality check of RNA immunoprecipitation (RIP) assay: analysis of LIN28B expression level by WB. The results of RIP assay by qRT-PCR of RIP materials for CDX2 (**B**), OCT4 (**C: upper**) and SOX2 (**C: lower**) (n=3) **(D)** mRNA stability assay in LoVo control and LIN28B overexpression. (n=3) Data are presented as mean ± SEM. Unpaired, two-tailed student's t-test (**B**-**D**) was performed. (*) $p < 0.05$, (**) $p<0.01$.

.

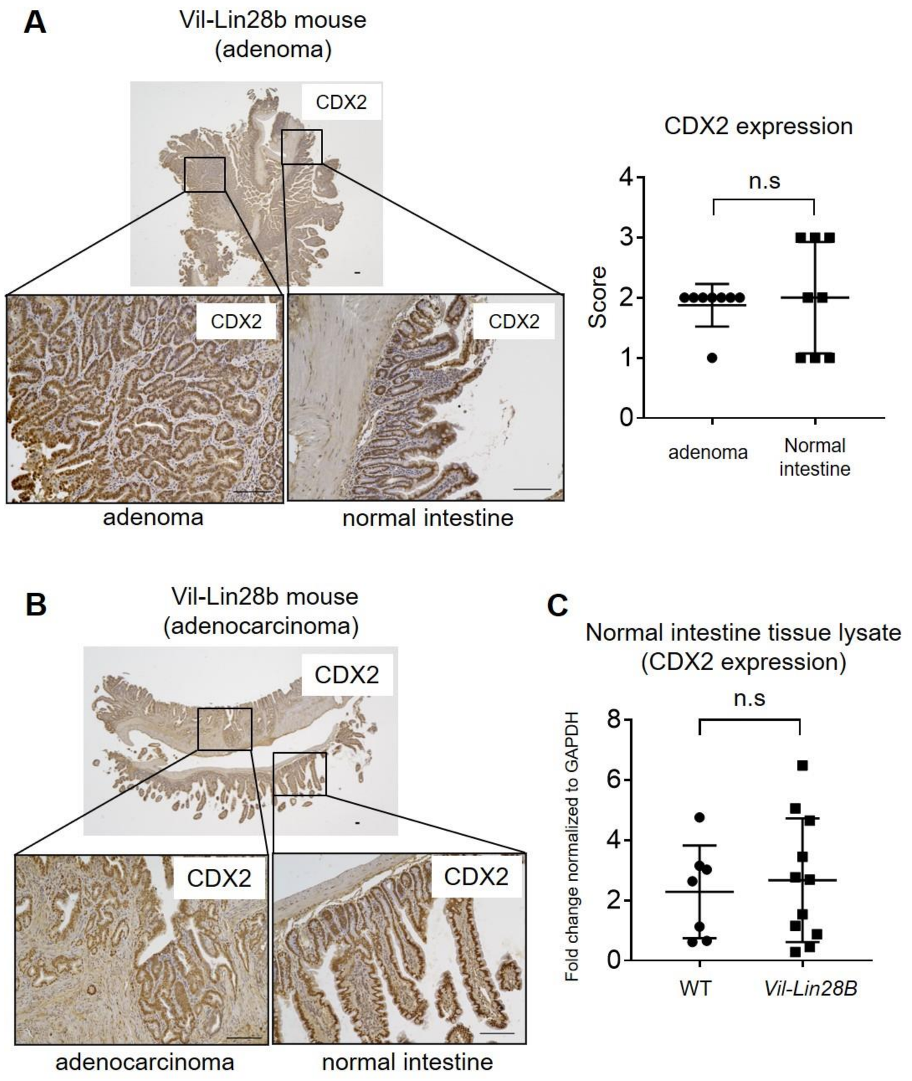

Supplemental Figure 3. CDX2 expression is maintained during tumorigenesis in LIN28B expressing transgenic mice. (A) Left: Representative IHC staining for CDX2 in an adenoma in *Vil-LIN28B* mice**; Right:** The score of CDX2 expression in adenomas and paired normal intestine in *Vil-LIN28B* mice. (n=8) **(B)** Representative IHC staining for CDX2 in an adenocarcinoma in *Vil-LIN28B* mice. **(C)** The graph indicates CDX2 expression in normal intestine of *Vil-LIN28B* mice (n=11) and WT (n=7) mice by western blotting analysis. (n=7) Data are presented as mean ± SD. Unpaired, two-tailed student's t-test (**A** and **C**) was performed.

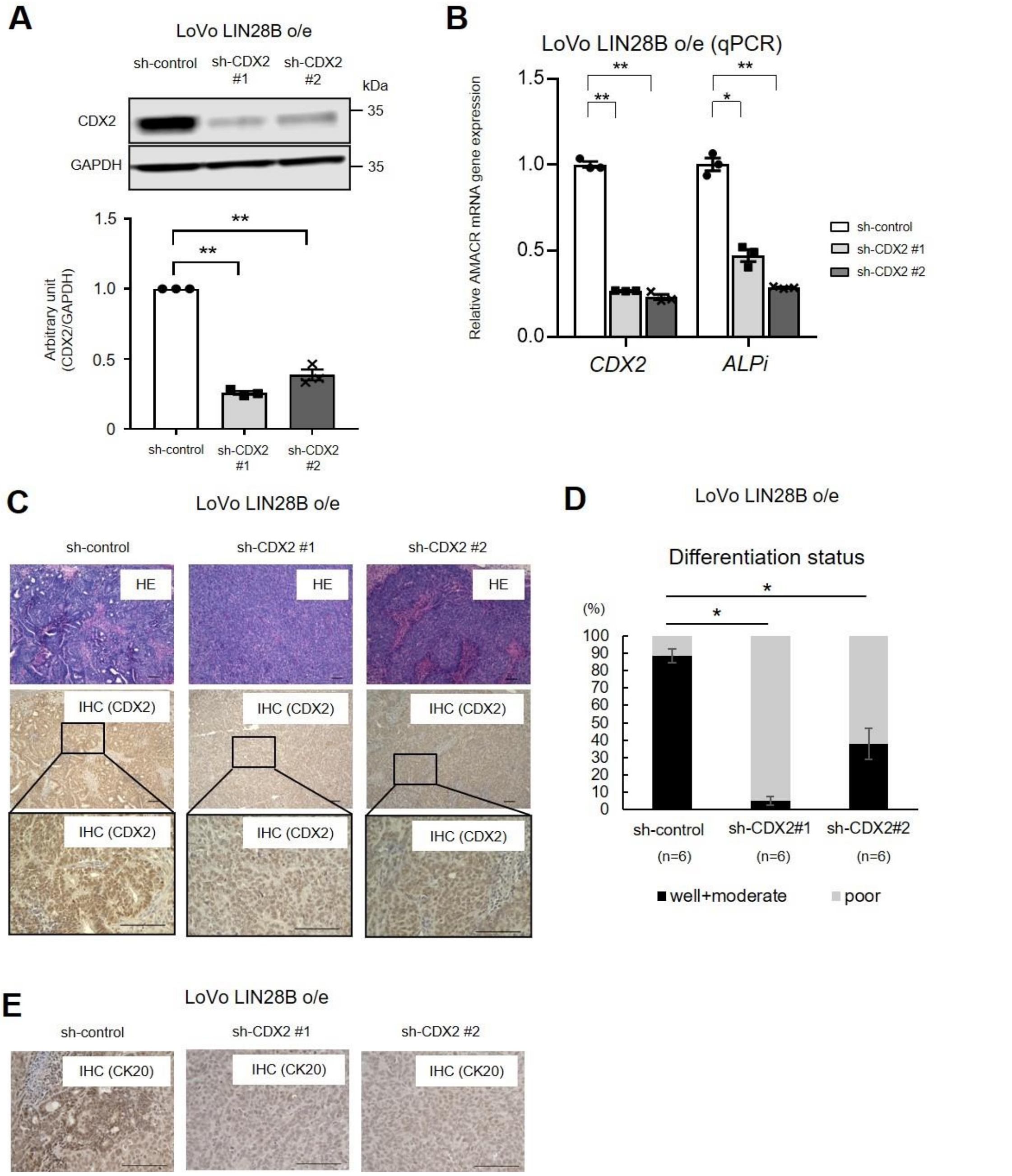

Supplemental Figure 4. CDX2 regulates CRC tumor differentiation in the context of LIN28B overexpression. (A) WB showing CDX2 knockdown using shCDX2 in LoVo cells. Lower graph shows the densitometry normalized by GAPDH. (n=3) **(B)** *CDX2* and *ALPi* expression (qPCR) in LoVo control/CDX2 KD cells (n=3). **(C)** Representative IHC in the subcutaneous xenograft tumor of LoVo cells with control or CDX2 KD for H&E (first panels), IHC of CDX2 (second and third panels). Scale bar, 100µm. **(D)** Cumulative ratio of differentiation status in subcutaneous xenograft tumors (n=6 per cell type). **(E)** Representative CK20 IHC in the subcutaneous xenograft tumor of LoVo cells with control or CDX2 KD. Scale bar, 100µm. Data are presented as mean ± SEM. One-way ANOVA followed by Dunnett's multiple comparisons test as post hoc analysis (**A**, **B** and **D**) was performed. (*) $p < 0.05$, (**) $p < 0.01$.

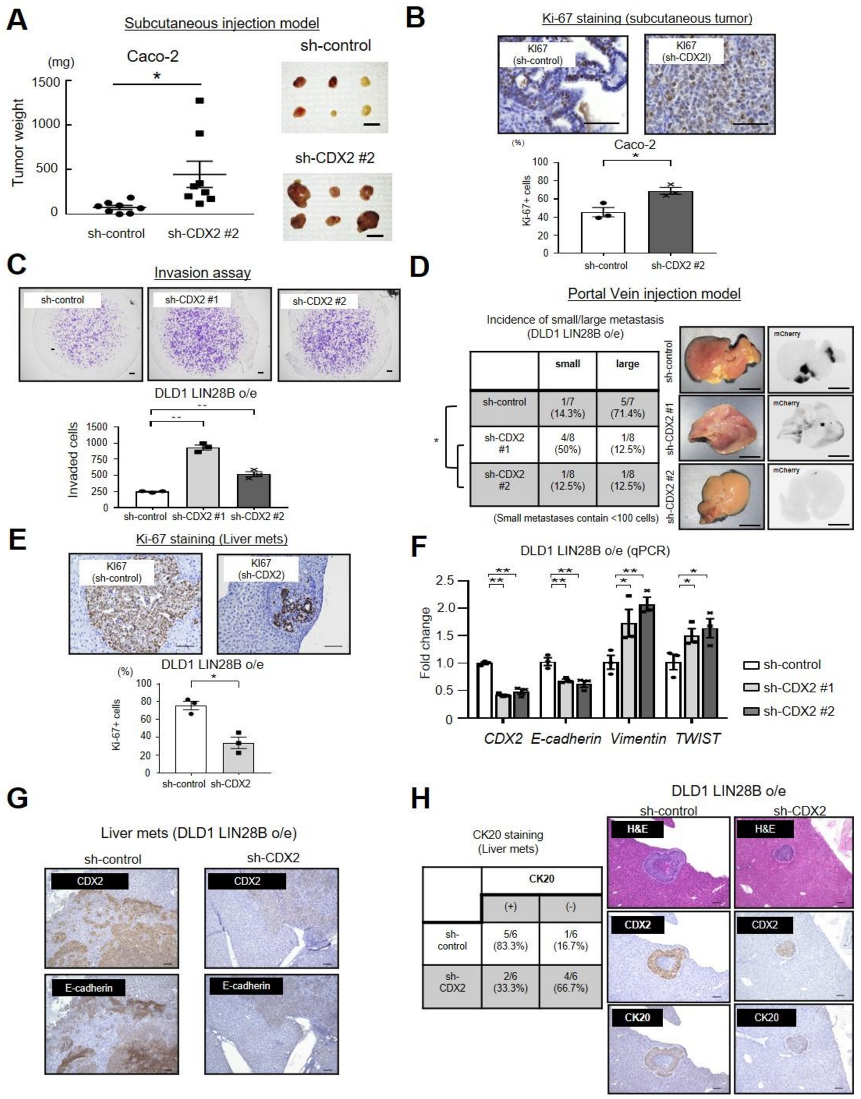

Supplemental Figure 5. CDX2 promotes metastatic CRC tumor colonization through mesenchymal-epithelial transition. (A) Left: Subcutaneous xenograft experiments with Caco-2 cells (n=6 per cell type) show a significant increase in tumor weight with CDX2 knockdown (447.00 ± 147.00 mg at sacrifice) as compared to control cells (75.63 ± 22.24 mg at sacrifice). **Right:** the images of tumors (Scale bar, 10 mm). **(B)** Ki-67 staining in the subcutaneous xenograft tumor of DLD1 cells with control or CDX2 KD, representative images (**upper**) and the quantification (**lower**). Scale Bars: 100μm. (n=3) **(C)** Transwell chamber invasion assay of DLD1 cells with control or CDX2 KD, representative images (**upper**) and the quantification (**lower**). Scale Bars: 100μm. (n=3) **(D) Left:** Frequency of small/large liver metastases in DLD1 control or CDX2 KD groups. **Right;** Representative liver images. (left: bright field, right: images with mCherry signal.) Scale Bars: 10mm. **(E)** Ki-67 staining in the liver metastatic tumor of DLD1 cells with control or CDX2 KD, representative images (**upper**) and the quantification (**lower**). Scale Bars: 100μm. (n=3) **(F)** Epithelial-mesenchymal transition (EMT) markers expression (qPCR) in DLD1 control/CDX2 KD cells (n=3). **(G)** Representative IHC of CDX2 and E-cadherin in the liver metastatic tumor of DLD1 cells with control or CDX2 KD. **(H) Left:** Frequency of CK20 positive liver metastases in DLD1 control or CDX2 KD groups. **Right;** Representative H&E and IHC staining (CDX2 and CK20). Scale Bars: 100μm. Data are presented as mean ± SEM.Unpaired, two-tailed student’s t-test (**A**, **B** and **E**), One-way ANOVA followed by Dunnett’s multiple comparisons test as post hoc analysis (**C** and **F**) or Chi-square test (**D**) was performed. (*) $p < 0.05$, (**) $p < 0.01$.

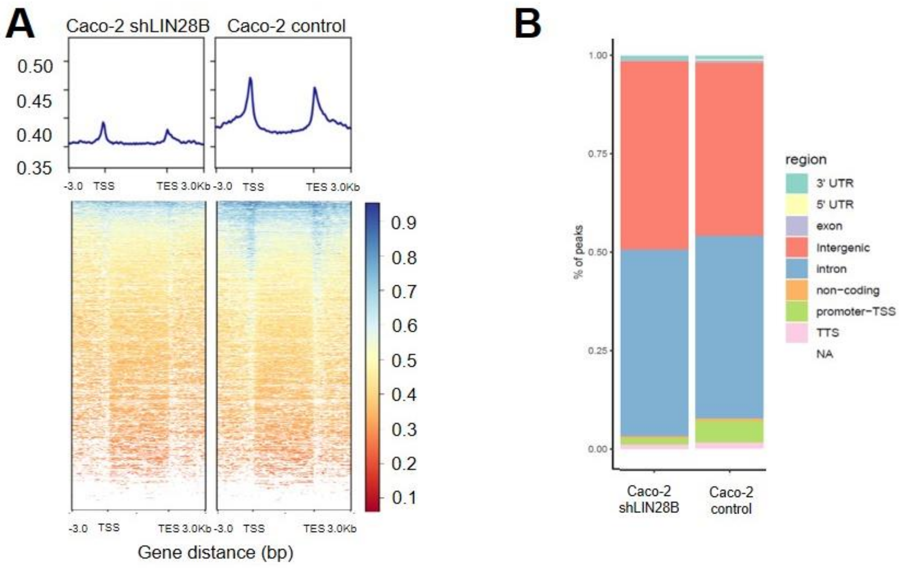

Supplemental Figure 6. CDX2 ChIP-seq identifies AMACR as a novel target for CDX2 in the context of LIN28B overexpression. (A) Heat maps of CDX2 ChIP-seq in Caco-2 cells with control or LIN28B knockdown. **(B)** The annotated site of Peak analysis in CDX2 ChIP-seq.

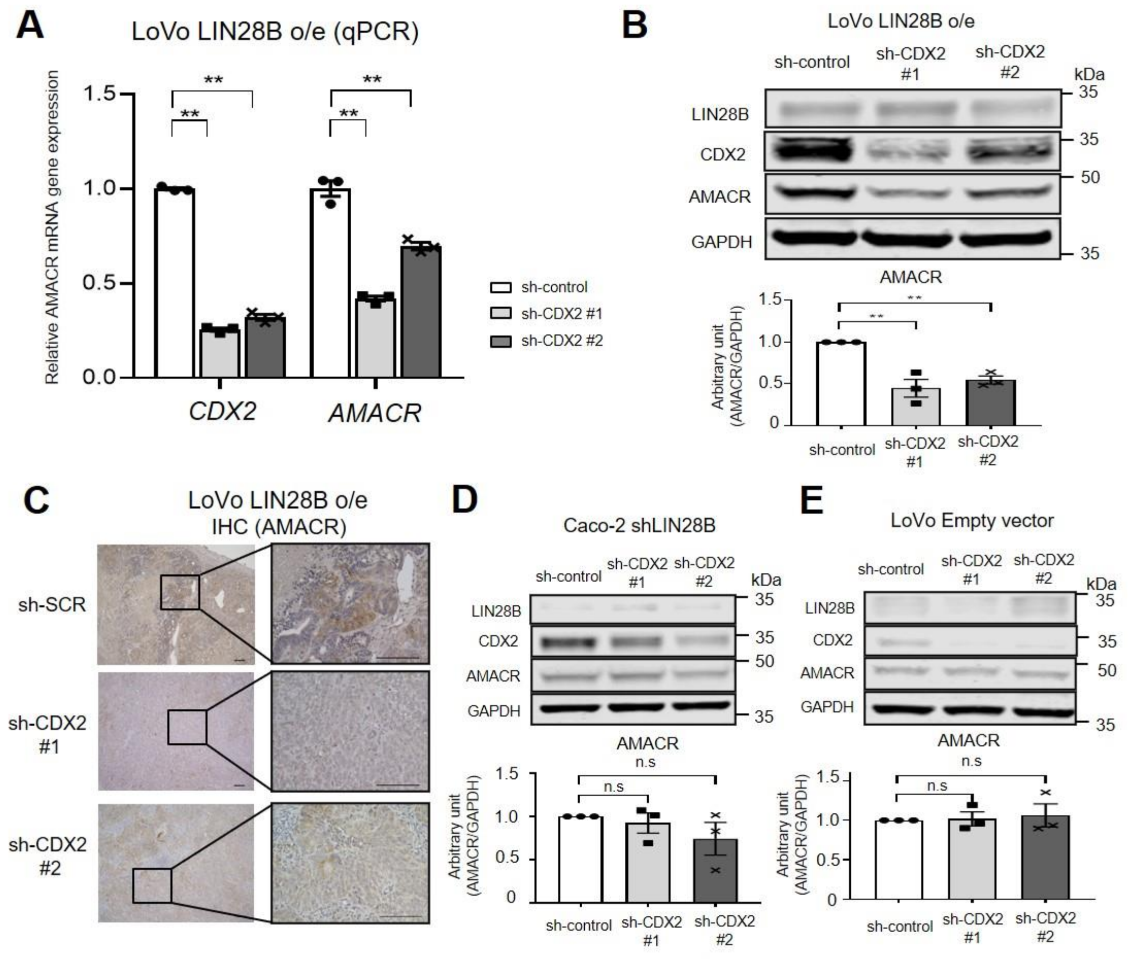

Supplemental Figure 7. CDX2 expression has a positive correlation with AMACR expression in CRC in the context of LIN28B overexpression. (A) *CDX2* and *AMACR* expression (qPCR) in LoVo LIN28B o/e and CDX2 KD cells. (n=3) **(B) Upper:** WB analysis of CDX2 and AMACR in LoVo LIN28B o/e and CDX2 KD cells. **Lower:** Band intensities were normalized by densitometry to GAPDH. (n=3) **(C)** Representative IHC staining for AMACR in the subcutaneous xenograft tumor of LoVo LIN28B o/e cells with control or CDX2 KD. Scale bars, 100µm. **(D) Upper:** WB analysis of CDX2 and AMACR in Caco-2 cell with both LIN28B and CDX2 KD. **Lower:** Band intensities were normalized by densitometry to GAPDH. (n=3) **(E) Upper:** WB analysis of CDX2 and AMACR in LoVo cells with control and CDX2 KD cells. **Lower:** Band intensities were normalized by densitometry to GAPDH. (n=3) Data are presented as mean ± SEM. One-way ANOVA followed by Dunnett's multiple comparisons test as post hoc analysis (**A**, **B**, **D** and **E**) was performed. (*) $p < 0.05$, (**) $p < 0.01$

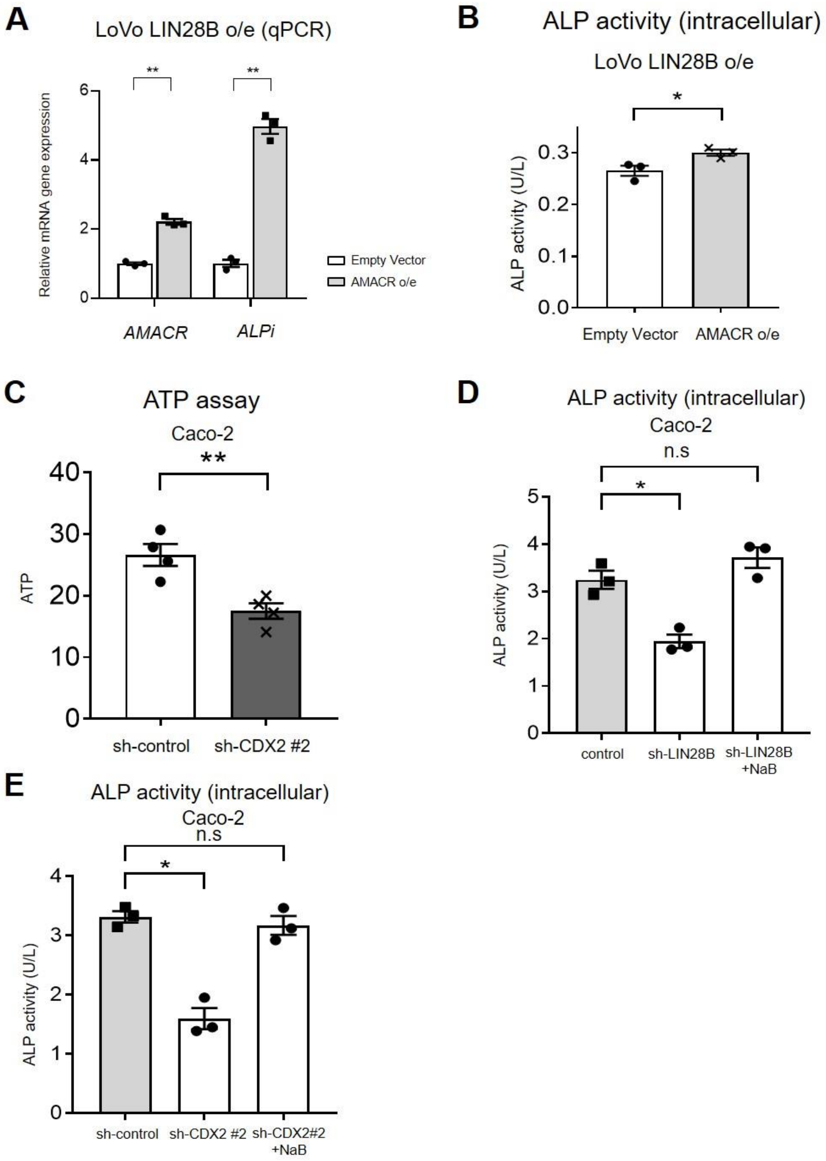

Supplemental Figure 8. AMACR enhances ALPi expression and activity in CRC cells in the context of LIN28B overexpression. (A) *AMACR* and *ALPi* expression (qPCR) in LoVo LIN28B o/e with Empty Vector/AMACR o/e. (n=3) **(B)** ALP activity assay in LoVo LIN28B o/e with Empty Vector/AMACR o/e. (n=3) **(C)** ATP assay in Caco-2 with CDX2 knockdown. (n=4) **(D)** ALP activity assay in Caco-2 control/LIN28B cells with/without sodium butyrate (Na-B). (n=4) **(E)** ALP activity assay in Caco-2 control/CDX2 KD cells with/without sodium butyrate (Na-B). (n=3) Data are presented as mean ± SEM.Unpaired, two-tailed student's t-test (**A**, **B** and **C**), One-way ANOVA followed by Dunnett's multiple comparisons test as post hoc analysis (**D** and **E**) was performed. (*) $p < 0.05$, (**) $p < 0.01$.

Supplemental Tables

Supplemental Table 1. Primer sequences for qPCR

hCDX2	Forw: CAGTCGCTACATCACCATCCG<br>Rev: TTTCCTCTCCTTTGCTCTGCG
hOCT4	Forw: GCCGGTTACAGAACCACACT<br>Rev: GTGGAGGAAGCTGACAACAA
hSOX2	Forw: TACAGCATGATGCAGGACCA<br>Rev: CCGTTCATGTAGGTCTGCGA
hAMACR	Forw: AAATGGTTATCATTAGGGCTTTTGA<br>Rev: TTCCTTTTTCACTAGAACCCATTCA
hGAPDH	Forw: TCAAGAAGGTGGTGAAGCAG<br>Rev: AAAGGTGGAGGAGTGGGTGT
hALPi	Forw: TGAGGGTGTGGCTTACCAG<br>Rev: TCCACGAAGAGGTAGAAG
hKLF4	Forw: CACCTGGCGAGTCTGACAT<br>Rev: GTCGCTTCATGTGGGAGAG
hMUC2	Forw: CAGCACCGATTGCTGAGTTG<br>Rev: GCTGGTCATCTCAATGGCAG
hATOH1	Forw: CCTTCCAGCAAACAGGTGAAT<br>Rev: TTGTTGAACGACGGGATAACAT
hNGN3	Forw: CTAAGAGCGAGTTGGCACTGA<br>Rev: GAGGTTGTGCATTCGATTGCG
hE-cadherin	Forw: TGCCCAGAAAATGAAAAAGG<br>Rev: GTGTATGTGGCAATGCGTTC
hVimentin	Forw: GAGAACTTTGCCGTTGAAGC<br>Rev: GCTTCCTGTAGGTGGCAATC
hTwist	Forw: GGAGTCCGCAGTCTTACGAG<br>Rev: TCTGGAGGACCTGGTAGAGG

Supplemental Table 2. Antibodies Used for IHC and Western Blots

**Antibodies for IHC**					
**Antigen/Gene**	**Species**	**Type**	**Source and Cat#**	**Dilution**	**Detection**
For in vivo experiments					
Human LIN28B	Rabbit	pAb	Cell Sig. Tech. #4196	1:1000	IHC
Mouse Lin28b	Rabbit	pAb	Cell Sig. Tech. #5422	1:500	IHC
CDX2	Rabbit	mAb	Abcam ab76541	1:1000	IHC
AMACR	Rabbit	pAb	Sigma HPA020912	1:20	IHC
CK20	Rabbit	pAb	Bioss bs-1588R	1:400	IHC
E-cadherin	Rabbit	mAb	Cell Sig. Tech. #3195	1:500	IHC
Ki-67	Mouse	mAb	BD Pharmingen	1:400	IHC
For human TMAs					
LIN28B	Rabbit	pAb	Sigma HPA061745	1:200	IHC
CDX2	Rabbit	mAb	Cell Marque #EPR2764Y	Prediluted	IHC
AMACR P504s(13H4)	Rabbit	mAb	Cell Marque # 504R	1:50	IHC
**Antibodies for Western Blots**					
**Gene**	**Species**	**Type**	**Source and Cat#**	**Dilution**	**Detection**
CDX2	Rabbit	mAb	Abcam ab76541	1:1000	near-IR Fluor.
LIN28B	Rabbit	pAb	Cell Sig. Tech. #4196	1:5000	near-IR Fluor.
CK20	Rabbit	pAb	Bioss bs-1588R	1:1000	near-IR Fluor.
AMACR	Rabbit	pAb	Sigma HPA020912	1:500	near-IR Fluor.
GAPDH	Mouse	mAb	Millipore Mab374	1:10,000	near-IR Fluor.
